# High-resolution quantitative T_2_ mapping of the human brain at 7 T using a multi-echo spin-echo sequence and dictionary-based modeling

**DOI:** 10.1162/IMAG.a.81

**Published:** 2025-07-25

**Authors:** Jochen Schmidt, Dvir Radunsky, Patrick Scheibe, Carsten Jäger, Noam Ben-Eliezer, Robert Trampel, Nikolaus Weiskopf

**Affiliations:** Department of Neurophysics, Max Planck Institute for Human Cognitive and Brain Sciences, Leipzig, Germany; International Max Planck Research School on Neuroscience of Communication: Function, Structure and Plasticity, Leipzig, Germany; Department of Biomedical Engineering, Tel Aviv University, Tel Aviv, Israel; Center of Neuropathology and Brain Research, Paul Flechsig Institute, Medical Faculty, University of Leipzig, Leipzig, Germany; Sagol School of Neuroscience, Tel Aviv University, Tel Aviv, Israel; Center for Advanced Imaging Innovation and Research (CAI2R), New-York University Langone Medical Center, New York, NY, United States; Felix Bloch Institute for Solid State Physics, Faculty of Physics and Earth System Sciences, Leipzig University, Leipzig, Germany; Wellcome Centre for Human Neuroimaging, Institute of Neurology, University College London, London, United Kingdom

**Keywords:** quantitative MRI, transverse relaxation, ultra high-field MRI, high-resolution T_2_ mapping, Bloch equation simulations, dictionary matching

## Abstract

Quantitative ${\text{T}}_{2}$ mapping offers a unique contrast for detailed brain imaging. At ultra-high field strengths (7 T), the higher signal-to-noise ratio (SNR) enables higher spatial resolution and the delineation of smaller structures. The translation of multi-echo spin-echo-based acquisitions to higher field strength, however, is complicated by inhomogeneities in the radio frequency (RF) transmit field resulting in stronger stimulated echoes and multi-echo refocusing pathways. The ${\text{T}}_{2}$ decay will thus depend on the specific sequence details and other experimental properties. The signal can be modeled by Bloch equation simulations to create a dictionary of possible signal patterns to fit the experimental data and estimate ${\text{T}}_{2}$. Particularly at smaller voxel sizes and shorter ${\text{T}}_{2}$ times, noise will affect the dictionary matching of the data by the introduction of a bias in the acquired signal magnitude dependent on the SNR. This study aims to develop a robust, accurate, and fast ${\text{T}}_{2}$ mapping approach at 7 T, addressing RF inhomogeneity and noise bias. We employed a 2D multi-echo spin-echo sequence combined with a Bloch equation simulation-aided dictionary matching technique. The method incorporated a pre-measured ${\text{B}}_{1}^{+}$ map for regularization of the dictionary fit and applied a patch-based PCA denoising algorithm with magnitude bias correction to mitigate noise-induced errors. The method was tested in simulations, phantom validations, and in five human participants. In vivo, ${\left(0.7\;mm\right)}^{3}$ isotropic high-resolution ${\text{T}}_{2}$ maps showed detailed contrast within cortical and subcortical areas. Notably, regions with high iron content, such as the substantia nigra or nucleus ruber, were distinctly visible. The proposed method provided consistent ${\text{T}}_{2}$ values across different brain regions that aligned well with the literature where available. Simulations and experiments demonstrated the importance of the noise correction to achieve high-quality maps. The proposed method can significantly contribute to studies on brain microstructure and pathology, since it produces reliable ${\text{T}}_{2}$ maps at high resolution.

## 1 Introduction

The acquisition of magnetic resonance imaging (MRI) data weighted toward the transverse relaxation time ${\text{T}}_{2}$ plays an important diagnostic role. Especially, the FLAIR (Hajnal et al., 1992) sequence (fluid attenuated inversion recovery) is one of the cornerstones of clinical MRI (Vargas et al., 2017). The high signal-to-noise ratio (SNR) and contrast-to-noise ratio (CNR) of ${\text{T}}_{2}$-weighted sequences are also exploited in neuroscientific studies (Trampel et al., 2011). However, such sequences are not only sensitive to ${\text{T}}_{2}$ relaxation but also influenced by the experimental setup (Pierpaoli, 2010) and offer a mixed contrast influenced by different physiological and technical parameters.

In contrast, quantitative mapping of relaxation parameters provides reproducible maps across time points, MR scanners, and MR protocols (Edwards et al., 2018; Leutritz et al., 2020; Weiskopf et al., 2013). Consequently, a reliable quantification of ${\text{T}}_{2}$ relaxation would facilitate the development of disease biomarkers (Bruschi et al., 2020). Furthermore, robust quantification of the ${\text{T}}_{2}$ value enables biophysical modeling of the MRI contrast and microstructure imaging, as ${\text{T}}_{2}$ was shown to correlate with iron and myelin content (Fukunaga et al., 2010).

However, due to the challenging nature of quantitative ${\text{T}}_{2}$ mapping, it is not routinely included in the more recent quantitative multi-parametric scanning approaches (Haacke et al., 2020; Weiskopf et al., 2013; Ye et al., 2021). Conventionally, quantitative ${\text{T}}_{2}$ is estimated from multiple spin-echo (SE) acquisitions with varying echo times ($T_{E}$) (Hahn, 1950). Minimizing the effects of diffusion on the signal acquired after long echo times is possible by implementing a train of refocusing pulses and reading out several echoes after one initial $90^{\circ }$ pulse (Oakden & Stanisz, 2014). The so-called Carr-Purcell-Meiboom-Gill (Carr & Purcell, 1954) (CPMG) or multi-echo spin-echo (MESE) sequence is not only less affected by diffusion but also allows for shorter overall scan times compared with SE methods.

Within an imaging voxel, a multitude of different tissue types might be present, leading to the assumption of multi-exponential ${\text{T}}_{2}$ decay characteristics (Dula et al., 2010; A. MacKay et al., 2006; A. L. MacKay & Laule, 2016). Decay characteristics further were found to show time-dependent behavior approaching a linear exponent only in the long time limit (Kiselev & Novikov, 2018). For simplicity and due to the demand of high number of echoes for multi-compartmental analysis, however, this work uses a simplification to a mono-exponential decay approximation per voxel, which is still a good approximation for cortical and subcortical structures:

$$S\left(T_{E}\right)=S_{0}\text{ exp}\left(-\frac{T_{E}}{T_{2}}\right)S_{1}\left(T_{1},\;T_{R}\right),$$ (1)

where $T_{R}$ is the repetition time, $S_{0}$ is the initial signal that depends on the sample magnetization per voxel, and system specifics such as the RF receive coils. $S_{1}$ is the signal attenuation caused by $T_{1}$ relaxation influences varying for different tissue.

Usually, a long $T_{R}>4\;\text{s}$ is used to minimize the influence of $T_{1}$ relaxation ($S_{1}(T_{1},\;T_{R})\to 1$ ) in equation 1). At $T_{R}=4.5\;\text{s},$ around 90% of the initial magnetization of WM and GM tissue ($T_{1}∼1.2-2.1\;\text{s}$ (Haast, 2018; Zhu et al., 2014)) contributions has recovered. Longer $T_{R}$ is preferable, which leads to relatively long scan times even for CPMG-based methods. The long scan times are even more pronounced when ${\text{T}}_{2}$ maps with very high spatial resolution are desired (Schmidt et al., 2021).

Non-SE-based ${\text{T}}_{2}$ mapping approaches, such as the steady-state-based DESPOT2 (Deoni et al., 2004), allow for much faster scanning but yield systematic offsets in estimated ${\text{T}}_{2}$ values (Teixeira et al., 2017) when compared with SE-based methods, such as MESE sequences. Another promising steady-state method, TESS (Heule et al., 2014), was also shown to produce reduced ${\text{T}}_{2}$ values when compared with SE acquisitions (Kraff et al., 2016). More recently, fast approaches based on the signal phase (Seginer & Schmidt, 2022) were used but also resulted in approximately 15−20% bias when compared with MESE values.

To a certain degree, MESE imaging can be accelerated by the use of parallel imaging (Blaimer et al., 2004) methods, like SENSE (Pruessmann et al., 1999) or GRAPPA (Griswold et al., 2002). Another promising approach is the implementation of joint echo reconstruction (Bilgic et al., 2018; Schmidt et al., 2023) via the usage of sparsity exploring reconstruction techniques such as LORAKS (Haldar, 2014). However, some of these advanced methods require special software and hardware. Thus, they are not straightforward to implement on conventional scanning platforms.

Additional challenges arise when translating ${\text{T}}_{2}$ mapping to higher field strengths which is necessary to achieve high spatial resolution. First, the energy of the radio frequency (RF) transmit (${\text{B}}_{1}^{+}$) field scales quadratic with field strength and even higher power is needed in temporal and cerebellar brain areas due to the spatially varying ${\text{B}}_{1}^{+}$ profile (Van de Moortele et al., 2005). As MESE sequences are based on the repetition of high RF amplitude refocusing pulses, the consequent high specific absorption rates (SAR) will limit their application in vivo and reduce the number of available echoes. Second, the inhomogeneity of the ${\text{B}}_{1}^{+}$ field is increased at 7 T (Lutti et al., 2012; Vaughan et al., 2001), causing the occurrence of stimulated echoes in a 2D sequence. Both effects complicate signal analysis, as the assumed simple exponential decay (see equation 1) model is not valid anymore. The limits of adapting this model, for example, by skipping more severely affected odd echoes, have been demonstrated (McPhee & Wilman, 2018).

At higher field strength, a more accurate signal model is necessary (Sled & Pike, 2000) and can be obtained by using the Extended Phase Graph approach (Hennig, 1988; Weigel, 2014) (EPG) or Bloch equation (Bloch, 1946) simulations (Ben-Eliezer et al., 2014). The former comes in a range of implementations (Guenthner et al., 2021; Malik et al., 2012), but it is important to incorporate ${\text{B}}_{1}^{+}$ effects on the slice profile especially when using 2D sequences (Lebel & Wilman, 2010; McRobbie et al., 1987; Wang et al., 2020). In the latter, the effect of ${\text{B}}_{1}^{+}$ offsets in the RF transmit field is incorporated across voxel positions and slice profiles inherently. Both, Bloch equation simulations and EPG, can be seen as forward models accounting for bias in the signal introduced by choices of scanning parameters and specifics of the scanning setup. It has been shown that Bloch equation simulation-based methods provide higher accuracy under various conditions compared with EPG approaches (McPhee & Wilman, 2016).

Estimation of ${\text{T}}_{2}$ using Bloch equation simulations requires the inversion of the forward model, which is not trivial (Stöcker et al., 2010). Recently developed methods such as magnetic resonance fingerprinting (Ma et al., 2013) are using the forward model to generate a dictionary. These dictionaries consist of simulated signal response curves for a given set of input parameters, used sequence parameters, and assumptions about the sample population. A dictionary matching approach, comparable with a grid search optimization, is used to fit the acquired data to the simulations and reconstruct the respective input parameters generating the closest matching signal response curve.

The echo-modulation curve (EMC) approach (Ben-Eliezer et al., 2015) is based on Bloch equation simulations of an MESE sequence and was used for accurate and reproducible ${\text{T}}_{2}$ mapping using an accelerated MESE sequence at 3 T (Radunsky et al., 2021). This technique was successfully applied at 7 T with a turbo-spin echo variant (Emmerich et al., 2018). Here, scan time reduction is achieved by the mixing of different echo k-space data, which also leads to relatively long echo spacing. Long echo spacing, however, might introduce bias in short ${\text{T}}_{2}$ component estimations and additionally encodes ${\text{T}}_{2}$ modulation within k-space dependent on the specific choice of ordering (Busse et al., 2008).

To avoid mixing of k-space data, we present a ${\text{T}}_{2}$ mapping approach that applies a standard MESE sequence at 7 T in combination with the EMC dictionary matching. As mentioned above, a key issue to be resolved is the problem of pronounced RF transmit field inhomogeneities at 7 T. The transmit field is one of the parameters in the Bloch equation simulation and part of the dictionary matching. Providing additional ${\text{B}}_{1}^{+}$ maps was found to increase ${\text{T}}_{2}$ estimation accuracy at 4.7 T (McPhee & Wilman, 2016). Therefore, we regularize the dictionary matching method with ${\text{B}}_{1}^{+}$ input from a dedicated measurement of the ${\text{B}}_{1}^{+}$ profile.

For high-resolution imaging, another potential source of bias stems from the low SNR due to the reduced voxel size. At higher field strength, the ${\text{T}}_{2}$ value of the tissue present in the human brain decreases (Emmerich et al., 2018; Schmidt et al., 2021). Especially signal acquired at long echo times or low ${\text{B}}_{1}^{+}$ saturation efficiency suffers from low SNR resulting in the signal approaching the noise floor. The effect of this noise bias on the EMC dictionary matching has not been studied, although the impact of noise characteristics on similar dictionary matching approaches is subject to current research (Hong et al., 2022). The original method used signal thresholding (Ben-Eliezer et al., 2014), which is not possible in our approach due to the low number of available echoes.

A straightforward approach to match noisy data to an idealized dictionary is the use of denoising algorithms in the pre-processing of the data (Balbastre et al., 2021; Moeller et al., 2021). Multi-echo data acquired with MESE sequences can be regarded as a time series per voxel and has redundancy in spatial information across echoes. Thus, a natural choice for denoising algorithms is exploiting spatial sparsity. As such, a denoising approach (Does et al., 2019) using local principal component analysis (PCA) has been shown to sufficiently improve data quality. In conjunction with PCA denoising methods, bias correction for magnitude noise offsets has previously been implemented (Manjón et al., 2015; Pieciak et al., 2018). Mitigating magnitude offsets is necessary to enhance dictionary matching accuracy and reproducibility independent of the used resolution and SNR (Stern et al., 2022).

In this study, we present a quantitative ${\text{T}}_{2}$ mapping approach at high-field 7 T with sub-millimeter isotropic resolution on phantom and in vivo data. We extend the EMC-based and sequence-simulation-aided dictionary modeling approach to an MESE sequence optimized with modified refocusing flip angles and echo spacing. Potential bias resulting from ${\text{B}}_{1}^{+}$ inhomogeneity and low SNR is corrected by additional input from a separate ${\text{B}}_{1}^{+}$ acquisition and patch-based denoising scheme, respectively.

## 2 Theory

This theory section briefly describes the dictionary fitting and denoising approach. In the following text, we will be using the relaxation rate ${\text{R}}_{2}$, instead of the frequently used relaxation time $T_{2}=1/R_{2}$.

### 2.1 Dictionary generation

The dictionary matching of the signal magnitude data is based on the EMC (Ben-Eliezer et al., 2014) approach. Using Bloch equation simulations to estimate how the specific MR sequence affects the spin isochromats, matrix propagation is applied with all parameters assumed to be dependent on their spatial configuration, that is, on their position ρ:

$$\text{M}\left(\rho ,t\right)={\left[M_{x}\left(\rho ,t\right),M_{y}\left(\rho ,t\right),M_{z}\left(\rho ,t\right),M_{0}\left(\rho \right)\right]}^{T}.$$ (2)

Assuming, without loss of generality, the external fields are of the form $\omega =-\gamma \;\text{B}\left(\rho ,t\right)={\text{e}}_{x}\omega_{x}\left(\rho ,t\right)+{\text{e}}_{y}\omega_{y}\left(\rho ,t\right)$ $+{\text{e}}_{z}\omega_{z}$, with ω being the Larmor frequency, we can rearrange the Bloch equations into its 4D matrix form:

$$\frac{\text{d}}{\text{d}t}\text{M}(\rho ,t)=\left(\begin{array}{cccc} -R_{2}\left(\rho \right) & \omega_{z}\left(\rho ,t\right) & -\omega_{y}\left(\rho ,t\right) & 0\\ -\omega_{z}\left(\rho ,t\right) & -R_{2}\left(\rho \right) & \omega_{x}\left(\rho ,t\right) & 0\\ \omega_{y}\left(\rho ,t\right) & -\omega_{x}\left(\rho ,t\right) & -R_{1}\left(\rho \right) & R_{1}\left(\rho \right)\\ 0 & 0 & 0 & 0 \end{array}\right)\text{M}(\rho ,t),$$ (3)

where $R_{1}$ and $R_{2}$ are the longitudinal and transverse relaxation rates, respectively. Following the original approach and not including $B_{0}$ inhomogeneities, eddy-current effects, gradient non-linearity effects, or hardware-specific inconsistencies in *read* and *phase* gradient directions and only considering slice gradients $G_{z}$, we arrive at the following representation:

$$\frac{\text{d}}{\text{d}t}\text{M}\left(\rho ,t\right)=\left(\begin{array}{cccc} -R_{2}\left(\rho \right) & \omega_{0}+\gamma \;\rho_{z}G_{z}\left(\rho ,t\right) & -\gamma \;B_{1,y}\left(\rho ,t\right) & 0\\ -\omega_{0}+\gamma \;\rho_{z}G_{z}\left(\rho ,t\right) & -R_{2}\left(\rho \right) & \gamma \;B_{1,x}\left(\rho ,t\right) & 0\\ -\gamma \;B_{1,y}\left(\rho ,t\right) & -\gamma \;B_{1,x}\left(\rho ,t\right) & -R_{1}\left(\rho \right) & R_{1}\left(\rho \right)\\ 0 & 0 & 0 & 0 \end{array}\right)M\left(\rho ,t\right),$$ (4)

with $\omega_{0}=-\gamma B_{0}$ generated by the main field of the scanner. The effects of the gradients $G_{z}\left(\rho ,t\right)$ and RF pulses $B_{1}\left(\rho ,t\right)$ within each time step evolution of a sequence can be computed using equation 4 under the assumption of a valid hard pulse approximation (Pauly et al., 1991; Subramanian et al., 1985) when the specific sequence parameters are known. Solving the above equation 4 for the individual small time steps $t_{i}$ of the sequence, we can compute the magnetization iteratively as

$${\text{M}}_{t_{i}}={\text{E}}_{t_{i}}\left(\Delta t_{i}\right)\text{M}\left(\rho ,t_{i-1}\right),$$ (5)

with a given initial magnetization ${\text{M}}_{0}\left(\rho ,t_{0}\right)$ and $\Delta t_{i}=t_{i}-t_{i-1}$ . ${\text{E}}_{t_{i}}$ is the small time step solution of the matrix in equation 4 under the hard pulse approximation. Hence, the signal response can be computed across one echo train as the magnitude of the sum of all transverse magnetization components at the given echo time $T_{E}$. Since we only consider signal variations in the slice direction, the sample is binned with a finite number of points $k=0,\ldots ,n_{s}$ along ρ z:

$$\theta \left(T_{E},{\text{M}}_{0},R_{1},R_{2},B_{1},G_{z}\right)={\left|\sum_{k=0}^{{\text{n}}_{\text{s}}}\prod_{t_{i}=0}^{T_{E}}{\text{E}}_{t_{i}}{\text{M}}_{t_{i}-1}\right|}_{x,y}$$ (6)

A dictionary of signal patterns θ can be modeled to represent the spin isochromats’ response to expected input parameters, assuming constant ${\text{R}}_{1}$, ${\text{R}}_{2}$, and ${\text{M}}_{0}$ across the sample. This model does not account for coil sensitivity or the transmit and receive characteristics of a multichannel RF system, beyond the ${\text{B}}_{1}^{+}$ bias.

### 2.2 ${\text{B}}_{1}^{+}$ modeling

The EMC approach was found to benefit from additional ${\text{B}}_{1}^{+}$ estimates at higher field strength (McPhee & Wilman, 2016). Hence, the dictionary matching was regularized by a measured ${\text{B}}_{1}^{+}$ map. Several ${\text{B}}_{1}^{+}$ mapping techniques are available in vivo at 7 T (Lutti et al., 2012; Sacolick et al., 2010; Yarnykh, 2006). Dependent on the choice of sequence parameters, tissue properties and ${\text{B}}_{1}^{+}$ variation, routinely used approaches have been demonstrated to show limtied dynamical range (Lutz et al., 2024). We decided to use the relatively fast AFI method to minimize overall scan time, however, the approach underestimates the ${\text{B}}_{1}^{+}$ profile in areas with low ${\text{B}}_{1}^{+}$ (Yarnykh, 2010). To not bias the ${\text{R}}_{2}$ estimation with inaccurate ${\text{B}}_{1}^{+}$ input, the regularizing ${\text{B}}_{1}^{+}$ input was modulated based on a confidence metric deduced from AFI data. The AFI method is based on the acquisition of two signal images ($S_{1,2}$ ) at different repetition times ($TR_{1,2}$ ). Given the AFI signal equation (Yarnykh, 2006):

$$B_{1}^{+}=\frac{100}{\alpha_{\text{set}}}\text{arccos}\left(\frac{r\nu -1}{\nu -r}\right),$$ (7)

where $\nu =TR_{2}\;/\;TR_{1}$ and $r=S_{2}\;/\;S_{1}$, an approximate error estimate can be calculated:

$$\sigma_{B1}^{2}={\left(\frac{\delta B_{1}^{+}}{\delta r}\right)}^{2}\sigma_{r}^{2}.$$ (8)

If the error in ν is taken to be negligible as well as neglecting correlations between $S_{1}$ and $S_{2}$, an approximate expression for $\sigma_{B1}^{2}$ can be obtained:

$$\sigma_{B1}^{2}=-{\left(\frac{100}{\alpha_{\text{set}}}\right)}^{2}\frac{\left(\nu -1\right)\left(\nu +1\right)}{\left(r-1\right)\left(r+1\right){\left(\nu -r\right)}^{2}}\sigma_{r}^{2}.$$ (9)

Similarly, we can get $\sigma_{r}$ from propagating through r,

$$\sigma_{r}=\frac{\sigma_{1}S_{2}+\sigma_{2}S_{1}}{S_{1}^{2}}.$$ (10)

Thus extracting $S_{1,2}$ and $\sigma_{1,2}$ from the respective AFI measurements, relative percentage error maps $\zeta \left(\rho \right)=100\;\sigma_{B1}\;/\;B_{1}^{+}$ can be calculated for each voxel ρ. The relative error is increasing for AFI ${\text{B}}_{1}^{+}$ regions with low ${\text{B}}_{1}^{+}$ values, that is, r→1 in equation 9. Since the EMC method also provides an estimate of ${\text{B}}_{1}^{+}$, a weighting based on the AFI relative error can be introduced to yield a regularized ${\text{R}}_{2}$ estimation by combining EMC and AFI ${\text{B}}_{1}^{+}$ estimates using an appropriate weighting factor η(ζ):

$$B1_{\text{REG}}^{+}\left(\rho \right)=\eta \left(\zeta \right)\;B1_{\text{AFI}}^{+}\left(\rho \right)+\left(1-\eta \left(\zeta \right)\right)\;B1_{\text{EMC}}^{+}\left(\rho \right).$$ (11)

The factor was designed to linearly decrease the weight of $B_{1,\text{AFI}}^{+}$ until an empirical error threshold $\zeta_{\text{th}}$ , which was determined by phantom measurements:

$$\eta \left(\zeta \right)=1-min\left(\frac{\zeta }{\zeta_{\text{th}}},1\right).$$ (12)

### 2.3 Noise modeling

Noise characteristics can be taken into account at the dictionary generation stage or when the matching of the data to the dictionary. The former leads to drastically increased dictionary sizes, which in turn cause higher computational demands. In the latter, maximum likelihood estimation (Doucette et al., 2021) might be used to find the optimal signal response. Given the small number of available echoes, both are ill-posed inversion problems. Hence, in our approach, the noise characteristics and consequent bias are treated in the pre-processing stage of the data.

Upon acquisition of MRI data via multichannel receive coil systems, magnitude coil combinations are usually done using Adaptive Combine (Walsh et al., 2000) or root Sum of Square (Roemer et al., 1990) methods. If the original complex-valued channel data noise is assumed to be uncorrelated and following a zero-mean Gaussian distribution, the combined magnitude data will follow a non-central chi distribution (Aja-Fernández & Vegas-Sánchez-Ferrero, 2016; Dietrich et al., 2008):

$$P\left(x;\;S,n,\sigma \right)=\frac{x^{n}}{\sigma^{2}\;S^{n-1}}\text{ exp}\left(\frac{-\left(x^{2}+S^{2}\right)}{2\sigma^{2}}\right)I_{n-1}\left(\frac{x\;S}{\sigma^{2}}\right),$$ (13)

where S is the true signal value, $I_{n-1}$ is the modified Bessel function of first kind, the parameter n is the number of independent channels, and σ is the assumed identical noise variance of the channels. For high SNR, equation 13 is described by a Gaussian-shaped function centered around S. For an SNR approaching zero, the mean of the distribution from equation 13 asymptotically approaches a non-zero value.

Since the signals of different receive channels might be correlated and are weighted by coil sensitivity profiles, the noise characteristics n and σ vary spatially and can be approximated by effective parameters (Aja-Fernández et al., 2014) $n_{\text{eff}}$ and $\sigma_{\text{eff}}$ . Thus, $n_{\text{eff}}$ and $\sigma_{\text{eff}}$ will generally vary across the image, that is, per neighborhood. For some channel combination methods in conjunction with parallel imaging, as used in SENSE, the factor $n_{\text{eff}}\sigma_{\text{eff}}^{2}$ becomes spatially invariant. For GRAPPA techniques, this is not the case if adaptive channel combination is used (Aja-Fernández & Vegas-Sánchez-Ferrero, 2016).

In general, especially for GRAPPA, additional information such as GRAPPA reconstruction weights is necessary for reliable noise characteristics estimation, which is typically not available for product reconstruction methods. To keep the processing and subsequent value analysis applicable to other MESE modalities, an estimation of $n_{\text{eff}}$ and $\sigma_{\text{eff}}$ based on the image background via the procedure outlined in St-Jean et al. (2020) is possible and the noise parameter estimates can be kept stationary across each image slice. This introduces bias especially in the noise variance estimates $\sigma_{\text{eff}}$ , as the noise properties become signal dependent. However, when using local or voxel-wise noise filtering methods, whose performance does not rely on the exact knowledge of the noise properties as long as noise is homogeneous across the neighborhood, the non-stationarity does not impose an issue (Aja-Fernández et al., 2014).

To denoise data, a patch-based PCA filtering scheme (Does et al., 2019) is used to leverage redundancies in the spatial information across echo images. Patches are formed from the signal of local voxel neighborhoods and across all available echoes. A threshold is used to separate signal carrying components from noise contributions within the singular values of the patch, which can be computed using considerations about the statistical distribution of noise components within singular value decompositions (Manjón et al., 2013; Veraart et al., 2016). A sliding window approach is used to recombine patches. The bias in the magnitude signal can then be corrected using the local noise estimates (Pieciak et al., 2018)

$$S_{\text{corr}}\left(i,t\right)={\left(\text{max\{}\sum_{j\in V\left(i,t\right)}w\left(i,j\right)\;S^{2}\left(j,t\right)-2n_{\text{eff}}\left(i\right)\;\sigma_{\text{eff}}^{2}\left(i\right),\;0\}\right)}^{\frac{1}{2}},$$ (14)

where V(i,t) is a sliding window of spatial patches containing all time series data of the neighboring voxels, and w(i,j) is the non-negative weight assigned to S(j,t) via comparison after the PCA thresholding, as outlined in Manjón et al. (2015). $n_{\text{eff}}$ and $\sigma_{\text{eff}}$ are considered to be constant across the neighborhood of voxel i.

## 3 Materials & Methods

### 3.1 Dictionary generation

The EMC approach was implemented as a package in Python 3.10 incorporating PyTorch (Paszke et al., 2019) to enable GPU accelerated computations (code available on GitHub (Schmidt & Scheibe, 2024)). A vendor-provided sequence simulation package (Siemens IDEA version VE12) was used to extract the exact sequence parameters, RF and gradient shapes and timings of the used MESE sequence. RF pulses were simulated as consecutive matrix multiplication according to their sampling times in the sequence as in equation 6. At each step within the echo train, a hard pulse approximation was used and relaxation impact was calculated. The simulation was carried out using 1000 isochromats across a slice extent of 10 mm , that is, a spatial resolution of 10 μm , using an equilibrium magentization vector per isochromat of ${\left(0,\;0,\;1,\;1\right)}^{T}$. The dictionary curves can be regarded as functions:

$$\theta \left(S_{0},T_{1},T_{2},B_{1}^{+},\alpha_{i},T_{E},G_{z},t\right)$$ (15)

where $\alpha_{i}$ is the flip angle of the $i^{\text{th}}$ RF pulse in the sequence.

The following parameter combinations were simulated. Given, $T_{1}\gg T_{2}$ at 7 T in the brain, the effect of $T_{1}$ on the echo modulation across an echo train was approximated with a uniform $T_{1}$ value of 1.5 s after estimating its influence on the pattern matching via simulations. The effect of finite $T_{R}$ is not captured in the simulation and might lead to incomplete equilibrium magnetization recovery. As mentioned in the theory section, we assume the influence of this effect on the modeling to be negligible for the tissue under consideration. For ${\text{T}}_{2}$ we used a wide range of values: [1, …, 60 ] ms in steps of 0.25 ms, [60, …, 120 ] ms in steps of 2 ms, and [120, … 600 ] ms in steps of 10 ms.

**Fig. 1.**
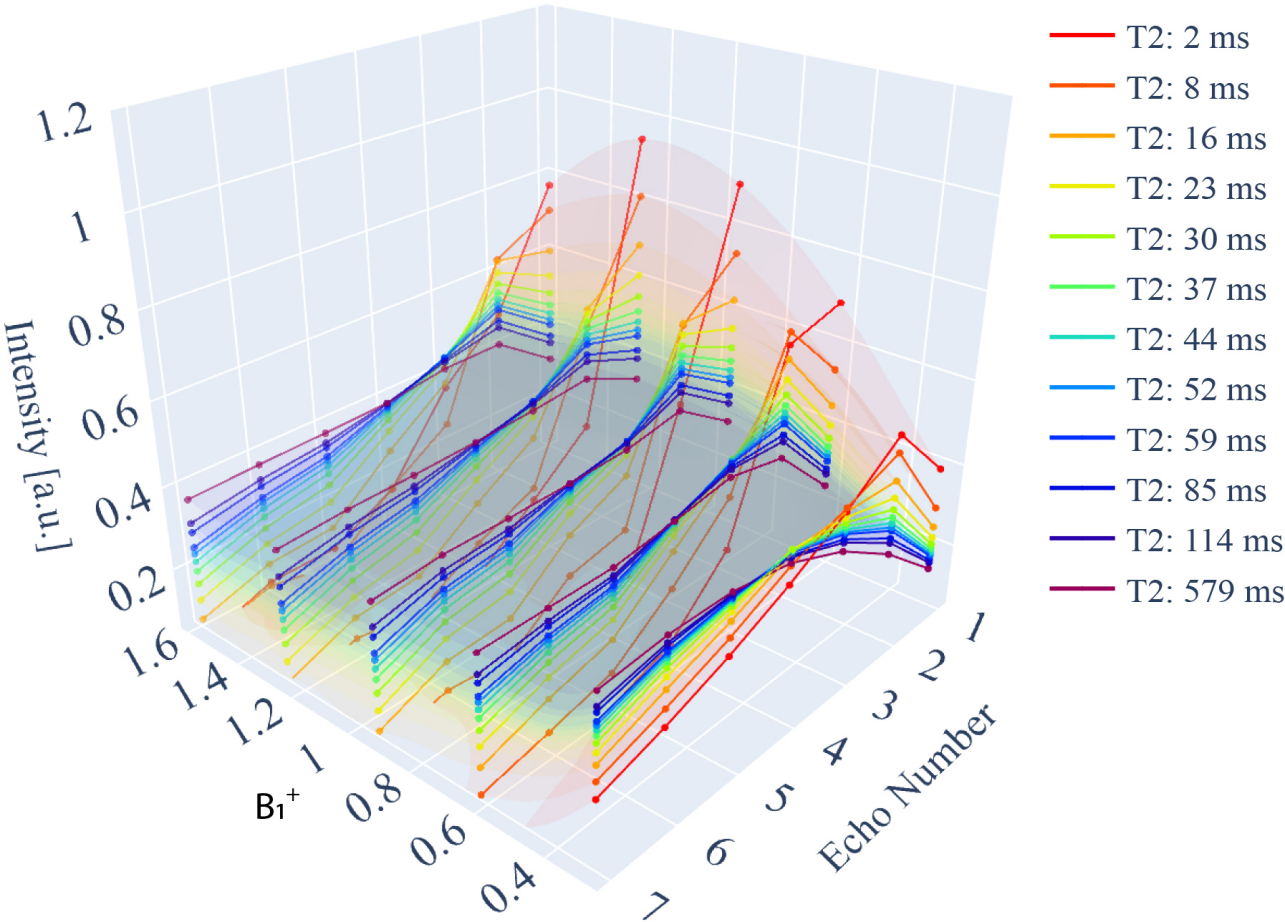
Exemplary dictionary of signal response curves. Input parameters contain among others the choice of sample ${\text{T}}_{2\;}$ and ${\text{B}}_{1}^{+}$ mismatch, leading to differences in the signal magnitude pattern simulated for the respective MESE sequence parameters at the respective echo time readouts.

The parameter choice was deduced from the range of ${\text{T}}_{2}$ values expected at 7 T for WM and GM areas. Due to the shorter echo train length, long ${\text{T}}_{2}$ times estimation was expected to be inaccurate, however, compartments exhibiting these values, like CSF, are not the primary focus of our study. For the normalized relative $B1_{\text{REG}}^{+}$ deviation, we used [0.2, … 1.6 ] in steps of 0.01 . The range of ${\text{B}}_{1}^{+}$ parameters for the simulation was driven by previous in vivo work (Lutti et al., 2012). Some of the simulated signal curves are plotted in Figure 1.

The dictionary of 44520 signal curves could be calculated within ∼40 min on a compute server with an NVIDIA Tesla V100 GPU with 32 GB RAM. The sequence parameters were kept constant across all acquisitions. Hence, the same dictionary could be used for fitting all data.

### 3.2 Noise modeling

The dictionary contains idealized signal evolutions for an acquisition with given sequence parameters. However, the acquired data will be influenced by noise, as well as a noise magnitude bias from channel combination. Consequently, a noise floor bias removal was adopted (Manjón et al., 2015) based on the MP-PCA local denoising procedure used in Does et al. (2019).

Patches for PCA were formed by collecting signal from quadratic 2D neighborhoods with side-length $l=\left\lceil \sqrt{ETL}\right\rceil$ across all echo data for each slice, where ETL is the echo-train length. Hence, we obtain $ETL\times N_{v}$ matrices, with $M=\text{min}\left(l^{2},N_{v}\right)=ETL$ from our choice of l. The signal mean across the spatial dimension $N_{v}$ is subtracted per patch and a singular value decomposition performed. A threshold is used to separate the highest P signal carrying components from remaining M−P noise contributions within the singular values. The “automated” rank cutoff calculation of P in the original MP-PCA approach introduced by Veraart et al. (2016) is strictly speaking not valid for real valued non-zero magnitude data. Hence, a fixed cutoff *P* = 2 was chosen as suggested in Does et al. (2019). After the filtering, a sliding window approach (Manjón et al., 2013) is used to combine the filtered patches.

The denoised signal can be bias corrected for the magnitude offset introduced by the non-central χ noise characteristics using equation 14. This assumed an effective $n_{\text{eff}}$ parameter of the distribution in equation 13 and the noise characteristics were assumed to be stationary across the image slice. The noise properties were deduced from an automated noise mask extraction (St-Jean et al., 2020), where the individual diffusion contrasts used in the original implementation were substituted by the individual echo magnitude images. The code was developed as a python package (Schmidt & Scheibe, 2024).

#### 3.2.1 Noise influence

We simulated dictionary matching performance with and without denoising and noise bias correction. A number of signal curves were chosen from the simulated dictionary. In total, 1000 noise samples were drawn from the distribution given in equation 13 for various parameters of $n_{\text{eff}}$ and $\sigma_{\text{eff}}$ , that is, different SNR, and added to the signal curves. The SNR was defined as the maximum signal intensity across the echo time series per dictionary curve, divided by the noise floor *mean*. Using the mean μ of the non-central χ distribution defined in equation 13, this gives

$$\text{SNR}=\underset{T_{E}}{\text{max}}S\left(\rho ,T_{E}\right)/\mu_{P(x|S=0,n_{\text{eff}},\sigma_{\text{eff}})}$$ (16)

Subsequently, we compared the fit performance of a dictionary matching approach, described later, with those noisy signal curves with and without bias correction. To validate the findings, we acquired phantom data with varying resolutions to effectively vary the voxel SNR of our measurements. The main insights of those studies informed the following *in vivo* studies and are briefly summarized here. We found magnitude bias correction to be necessary for accurate high-resolution ${\text{R}}_{2}$ estimation, especially for low-SNR voxels. We found that signal below an SNR of 5 cannot be reliably corrected with the proposed method. To mitigate the influence of those low-SNR voxel time series data, we used a threshold in all further analyses. All voxels with an SNR below 5 were excluded from the subsequent analysis.

### 3.3 Data acquisition

#### 3.3.1 Phantom

A commercially available phantom (Gold Standard Phantoms, Multi-Sample 120, Rochester, UK) containing 24 vials with a solution of various ${\text{MnCl}}_{2}$ concentrations was used for the measurements. The phantom consists of a cylindrical sample holder with concentrically placed vials. The ${\text{MnCl}}_{2}$ concentration ranged from 0.017 to 0.63 mM . The concentrations of the respective vials and their positions are detailed in the Supplementary Material (S1). All concentrations are present in two separate vials to assess the robustness and consistency of the method and positional dependency on ${\text{B}}_{1}^{+}$. After processing of the data, values were extracted from the phantom data by placing circular regions of interest (ROIs) in all vials and subsequently taking the mean of the voxel ${\text{R}}_{2}$ estimates within ROIs of vials with equal ${\text{MnCl}}_{2}$ concentrations.

#### 3.3.2 Participants

In total, five participants (three females, age 29.6±7.16 y [mean ± standard deviation]) were scanned using the MESE and AFI acquisitions described below. For anatomical reference, an MP2RAGE (Marques et al., 2010) product sequence was acquired. One subject was scanned twice to evaluate scan–rescan stability and reproducibility of the method. The study was approved by the ethics commission at the Faculty of Medicine of Leipzig University and written informed consent was obtained.

#### 3.3.3 MR sequence parameters

All data were acquired on a Magnetom Terra 7 T MRI scanner (Siemens Healthineers, Erlangen, Germany) using an 8Tx/32Rx channel phased array RF head coil (Nova Medical, Wilmington MA, USA). The transmit mode was set to Siemens “True Form” to produce a circularly polarized (CP) ${\text{B}}_{1}^{+}$ transmit field. To assess the accuracy of the method, the phantom was additionally scanned on a Prisma 3 T MRI Scanner (Siemens Healthineers, Erlangen, Germany) using a 32-channel receive RF head coil and the MESE sequence detailed in the following section.

##### 3.3.3.1 MESE

A 2D slice-selective MESE sequence was used. We used a long repetition time ($T_{R}=4500\;\text{ms}$ ) to prevent saturation from incomplete longitudinal relaxation and to minimize $T_{R}$ influence on $R_{2}$ estimation (Birkl et al., 2020). A 2D sequence offers lower SAR and high within-slice $T_{R}$. The settings allowed to measure in vivo with an SAR rating of about 80% for the head allowing for inter-individual variations.

An isotropic resolution of ${\left(0.7\;\text{mm}\right)}^{3}$ was achieved for all in vivo scans. To validate noise influence simulations, phantom measurements were also conducted at resolutions ${\left(0.9\;\;\text{mm}\right)}^{3}$ and ${\left(1.1\;\;\text{mm}\right)}^{3}$, the former was also used in the 3 T validation scans. The in vivo scans used an FOV of 212 mm×163.2 mm×72.1 mm , with a matrix size of 304×234×35 , acquiring 35 axial slices in interleaved order. The limited slice coverage (24.5 mm ) was due to high SAR demands, necessitating a 200% gap between slices to acquire data ranging from lower parts of the temporal lobes to the parietal lobe.

Note that the voxel resolution of the presented data remains unchanged by the choice of the slice gaps. It is determined by the slice profile of the pulse and the imaging gradients within the slice acquisitions. Magnetization transfer effects on ${\text{R}}_{2}$ were found to be unaffected by the choice of the slice gap (Radunsky et al., 2019).

The echo times were adjusted as follows: $T_{E}=\text{esp}*k$ with k=1, 2,…, 7 . For the 3 T scans, an echo spacing of esp=10.2 ms was used. The scanning at 7 T was done using a short echo spacing of 9 ms , necessary due to the longer ${\text{R}}_{2}$ (Wiggermann et al., 2020), especially when mapping subcortical structures (Miletić et al., 2020). We achieved short echo spacing by minimizing the duration of spoiler gradients straddling the RF refocusing pulses ($t_{\text{spoil}}=1.35\;\text{ms}$ ). The spoiling gradients are set such that a CPMG style sequence scheme is achieved. The overall short echo train length is necessary to achieve feasible scan time and to achieve a higher number of slices within one $T_{R}$. However, this is at the expense of estimation accuracy for low $\;{\text{R}}_{2}$ compartments, like CSF, which is not the primary focus of this study. The minimum moment necessary for the spoiler gradients was determined by EMC simulations as well as visual investigation of the images and chosen to be ∼55 mT.ms / m .

A bandwidth of 280 Hz / pixel was used to keep the readout time short while retaining sufficient SNR (Bernstein et al., 2010). Parallel imaging was used to speed up scans using GRAPPA under-sampling with an acceleration factor of 2, using 36 reference lines. This resulted in a total scan time of 10:12 min . Higher acceleration factors led to reconstruction errors (Schmidt et al., 2023). To minimize SAR, the MESE sequence used refocusing pulses of only $140^{\circ }$ instead of $180^{\circ }$. The effect of the lower flip angle is taken into account in the dictionary simulation (see Emmerich et al. (2018)).

##### 3.3.3.2 MP2RAGE

For anatomical reference, an MP2RAGE (Marques et al., 2010) dataset was acquired using the following parameters: $T_{R}=6000\;\text{ms}$ ; $T_{E}=2.06\;\text{ms}$ ; $T_{I}1\;/\;2=800\;/\;2700\;\text{ms}$ ; $\text{FA}1\;/2=4^{\circ }\;/\;5^{\circ }$; BW=240 Hz / pixel ; ${\left(0.63\;\text{mm}\right)}^{3}$ isotropic resolution. The FOV was set to 240×240×181 and the corresponding matrix size was $384\times 384\times 288\;{\text{mm}}^{3}$. A 4.6× -accelerated compressed sensing under-sampling scheme was applied to speed up acquisition covering (Ferraro et al., 2022). The resulting acquisition time was 8:40 min .

##### 3.3.3.3 AFI

The ${\text{B}}_{1}^{+}$ mapping used the AFI method (Yarnykh, 2006) with an isotropic resolution of ${\left(3\;\text{mm}\right)}^{3}$, as ${\text{B}}_{1}^{+}$ was assumed to vary smoothly across the sample. The sequence used parameters following the work of Lutti et al. (2012): $T_{R}1\;/\;2=16\;/\;160\;\text{ms}$ ; $T_{E}=3.08\;\text{ms}$ ; $\text{FA}=60^{\circ }$; BW=425 Hz / pixel . Diffusion spoiling moments were adopted to our scanning system and 216 ​/​ 1080 mT.ms / m was used, which is about three-fourth of the original setting. As in Lutti et al. (2010), spoiling along the read direction with 26 ​/ ​78 mT.ms ​/ ​m was used.

### 3.4 Data analysis

#### 3.4.1 Gibbs ringing artifact removal

The low-resolution phantom dataset (using ${\left(1.1\;\text{mm}\right)}^{3}$ isotropic resolution) exhibited Gibbs ringing artifacts, attributed to the geometric characteristics of the vials. Preceding data modeling, a Gibbs artifact mitigation based on local sub-voxel displacements, was used (Kellner et al., 2015).

#### 3.4.2 $B_{1}^{+}$ processing

An estimate of $B1_{EMC}^{+}$ was obtained using the dictionary matching without any ${\text{B}}_{1}^{+}$ regularization (see equation 17 below). The resulting map was smoothed by a small Gaussian filter, using SciPy (Virtanen et al., 2020) with a sigma kernel of five voxels in each direction, as it shows areas of high variation between neighboring voxels but ${\text{B}}_{1}^{+}$ is assumed to be varying slowly spatially.

The individual AFI signal echo images ($S_{1,2}$ ) were used in two ways. First, the data were processed using the hMRI-toolbox (Tabelow et al., 2019) as a module of SPM12 (Friston et al., 2007) implemented in MATLAB (The MathWorks Inc., 2022). The resulting ${\text{B}}_{1}^{+}$-reference volume was co-registered and resampled to the first echo of the MESE signal data using ANTs (Tustison et al., 2014) via an affine transform and b-spline interpolation. Second, noise properties were extracted using the same automated noise mask extraction (St-Jean et al., 2020) and fit to equation 13 to extract $\sigma_{1,2}$ . In turn, equation 9 can be used to extract an expression for $\sigma_{B1}$ which was used to estimate a relative percentage error map ζ(ρ) for each voxel ρ. Subsequently, the $B1_{AFI}^{+}$ estimate and the relative percentage error map ζ(ρ) were registered and re-sampled using the above transform and linear interpolation.

The threshold $\zeta_{\text{th}}$ for the weighting in equation 11 was empirically determined from phantom measurements by minimizing the coefficient of variation of the ${\text{R}}_{2}$ estimates within vials of identical ${\text{MnCl}}_{2}$ concentration. The $B1_{REG}^{+}$ input for regularization of the dictionary matching was obtained using equation 11 with the estimates of $B1_{EMC}^{+}$ and $B1_{AFI}^{+}$.

#### 3.4.3 Anatomical processing pipeline

##### 3.4.3.1 Registration

All resulting maps were skull stripped via fsl-bet (Jenkinson et al., 2012). The anatomical reference scan was co-registered to the MESE echo data using ANTs (Tustison et al., 2014) via TRSAA transform. The transform is calculated using mutual information maximization for translation, rigid movement and affine transforms between the data. We used the second inversion image of the MP2RAGE data and the $S_{0}$ estimate (see below) of the MESE data for co-registration due to contrast similarity. The transform was used for subsequent processing.

##### 3.4.3.2 Segmentation

The acquired MP2RAGE data were used for segmentation of the brain via the FreeSurfer (Dale et al., 1999) script recon-all-clinical (Billot, Magdamo, et al., 2023; Gopinath et al., 2023; Iglesias et al., 2023) implementing SynthSeg (Billot, Greve, et al., 2023) to segment the MP2RAGE UNI images. The obtained atlas-based labels (Desikan et al., 2006) were used in multiple ways.

##### 3.4.3.3 Extraction of mid cortical layer

A combined image was produced to get a homogeneously delimited gray matter (GM) band. This image was up-sampled to (0.3 mm)^3^ isotropic resolution using nearest neighbor interpolation and put into the LayNii (Huber et al., 2021) function LN2_RIMIFY to get an input for LayNii LN2_LAYERS to compute three cortical layers. The mid layer was transformed to MESE data space and resampled using b-spline interpolation. Cortical mid-layer correspondence was obtained via thresholding. Only voxels with values above 0.75 were considered part of the mid GM layer.

##### 3.4.3.4 Parcellation of cortical areas

The labels were combined as described in Klein and Tourville (2012) to define six cortical ROI (frontal lobe, parietal lobe, temporal lobe, occipital lobe, cingulate cortex, insula). The ROIs were inspected and manually corrected with help of the MP2RAGE UNI and T1-weighted images. ${\text{R}}_{2}$ values were attributed to the respective structures based on the obtained labels and above GM cortex definition.

##### 3.4.3.5 Definition of subcortical structures

The SynthSeg-based segmentation performed poorly in subcortical and brain stem regions, and other automatic segmentation methods of the subcortical areas, such as MASSP (Bazin et al., 2020), could not be applied due to the need of additional contrast scans like $R_{2}^{*}$. Hence, besides manual correction of the automated segmentation, subcortical ROIs such as substantia nigra and nucleus ruber were labeled manually by a trained neuroanatomist, with 15 years of experience. For anatomical reference, data from the Human Brain Atlas (Mai et al., 2011) were used. Subsequently, all obtained label volumes were registered onto the individual MESE data using the same transform as the MP2RAGE images.

##### 3.4.3.6 Eroding of parcellated areas

All labels were processed by binary erosion with a small 3D voxel kernel of radius r=1 using SciPy (Virtanen et al., 2020). This way partial volume effects were reduced by shrinking the labeled ROI volumes, since fine-grained delineation of region boundaries and comprehensive coverage of the entire ROI were less important for this study.

#### 3.4.4 Dictionary matching

We used an optimization procedure minimizing the L2 norm of the difference between acquired data and dictionary entries, while regularizing the ${\text{B}}_{1}^{+}$ parameter with the $B_{1,\text{REG}}^{+}$ estimate informed by the AFI acquisitions. The dictionary curves θ (see equation 6) were normalized after creation via an L2 norm across the time dimension ($\rho_{\theta }\left(\theta \right)$). Input signal curves were also L2 normalized across the echo time dimension giving a roughly SNR-based scaling parameter $\rho_{S}\left(S\left(\rho ,t\right)\right)$. The matching was done with normalized curves. Without L2 normalization of both, the matching would be signal intensity dependent. Other normalization strategies, such as normalizing by maximum value within the signal echo train, or the use of the dot product between the curves, were not found to affect matching performance. However, in the former, the introduction of a small scaling parameter to mitigate noise influence on the single maximum signal echo value was previously used (Doucette et al., 2021).

$$\underset{T_{2}\left(\rho \right),B_{1}^{+}\left(\rho \right)}{\text{min}}{\left\|\frac{S\left(\rho ,t\right)}{\rho_{S}\left(\rho \right)}-\frac{\Theta \left(T_{2},B_{1}^{+},t,\;.\;.\;.\right)}{\rho_{\theta }\left(T_{2},B_{1}^{+}\right)}\right\|}_{L2}+\lambda {\left\|\;B_{1,\text{REG}}^{+}\left(\rho \right)-B_{1}^{+}\left(\rho \right)\;\right\|}_{L2}.$$ (17)

Successively, after matching the dictionary entries, $\rho_{S}\left(\rho \right)\;/\;\rho_{\theta }\left(T_{2}\left(\rho \right),B_{1}^{+}\left(\rho \right)\right)\approx S_{0}$ could be used as an approximation of the input signal intensity. This estimate was found to give accurate registration results and was subsequently used in the processing pipeline.

#### 3.4.5 $R_{2}\;$ value analysis

**Fig. 2.**
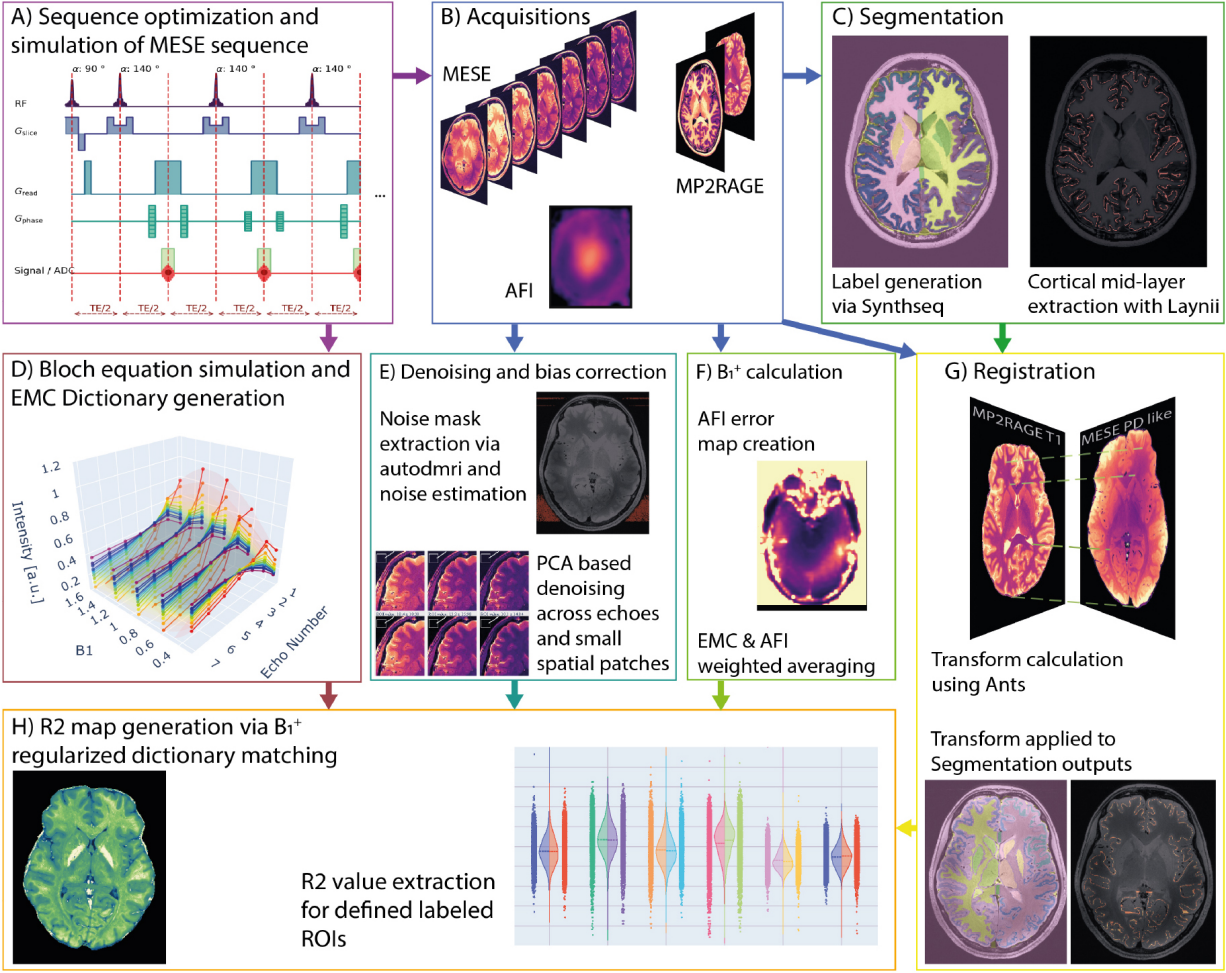
Overview of the whole pipeline and data flow. First (A) the multi-echo spin-echo (MESE) product sequence was optimized for echo spacing and low SAR and subsequently simulated via a vendor-provided sequence simulation tool. We then used the sequence to acquire MESE echo data (B) and the simulation output to inform the Bloch equation simulation for dictionary creation (D). Additionally, MP2RAGE (Ferraro et al., 2022; Marques et al., 2010) was acquired for anatomical reference and actual flip angle (AFI) imaging (Lutti et al., 2012; Yarnykh, 2006) mapped the ${\text{B}}_{1}^{+}$ profile. We extracted ${\text{B}}_{1}^{+}$ profiles and error maps from the AFI acquisitions (F) to calculate a ${\text{B}}_{1}^{+}$ map as input for dictionary matching regularization. The resulting ${\text{B}}_{1}^{+}$ map was resampled to fit the MESE matrix. The acquired MP2RAGE data were used as input for the segmentation (C) and cortical mid-layer extraction using LayNii (Huber et al., 2021). We registered the MP2RAGE data onto the MESE signal intensity estimate (G) using ANTsPy (Tustison et al., 2014) to transform the segmented labels and cortical definitions into the MESE data space. All MESE echo images were denoised with a PCA denoising scheme (Does et al., 2019) after noise estimation (E) using autodmri as described in St-Jean et al. (2020). Noise bias correction was applied to the denoised data (E). The resulting echo data as well as the resampled ${\text{B}}_{1}^{+}$ map were used for dictionary matching (H) to produce ${\text{R}}_{2}$ maps. Combined with the labels obtained (C and G), we then proceeded to determine mean values and distributions for each ROI.

We used the labels co-registered onto the MESE data to extract the ${\text{R}}_{2}$ voxel values in each ROI. The whole pipeline was used for each volunteer scan and the average across all participants was determined. Figure 2 depicts the whole processing pipeline. For scan–rescan evaluation in the single volunteer who was scanned twice, we provided the Pearson correlation coefficient for the ROI mean ${\text{R}}_{2}$ values and Bland–Altman statistics and the coefficient of variation.

## 4 Results

### 4.1 Simulations

The noise simulations showed systematically inflated magnitude values at low SNRs. The simulated magnitude data asymptotically approached the noise floor mean value. While the PCA denoising appears to reduce the spread in individual noise values, the noise mean bias is unchanged. Including the bias correction factor (equation 14) reduces the deviation from the ground truth except for very low SNR. The known noise distribution characteristics were used in the bias correction approach.

**Fig. 3.**
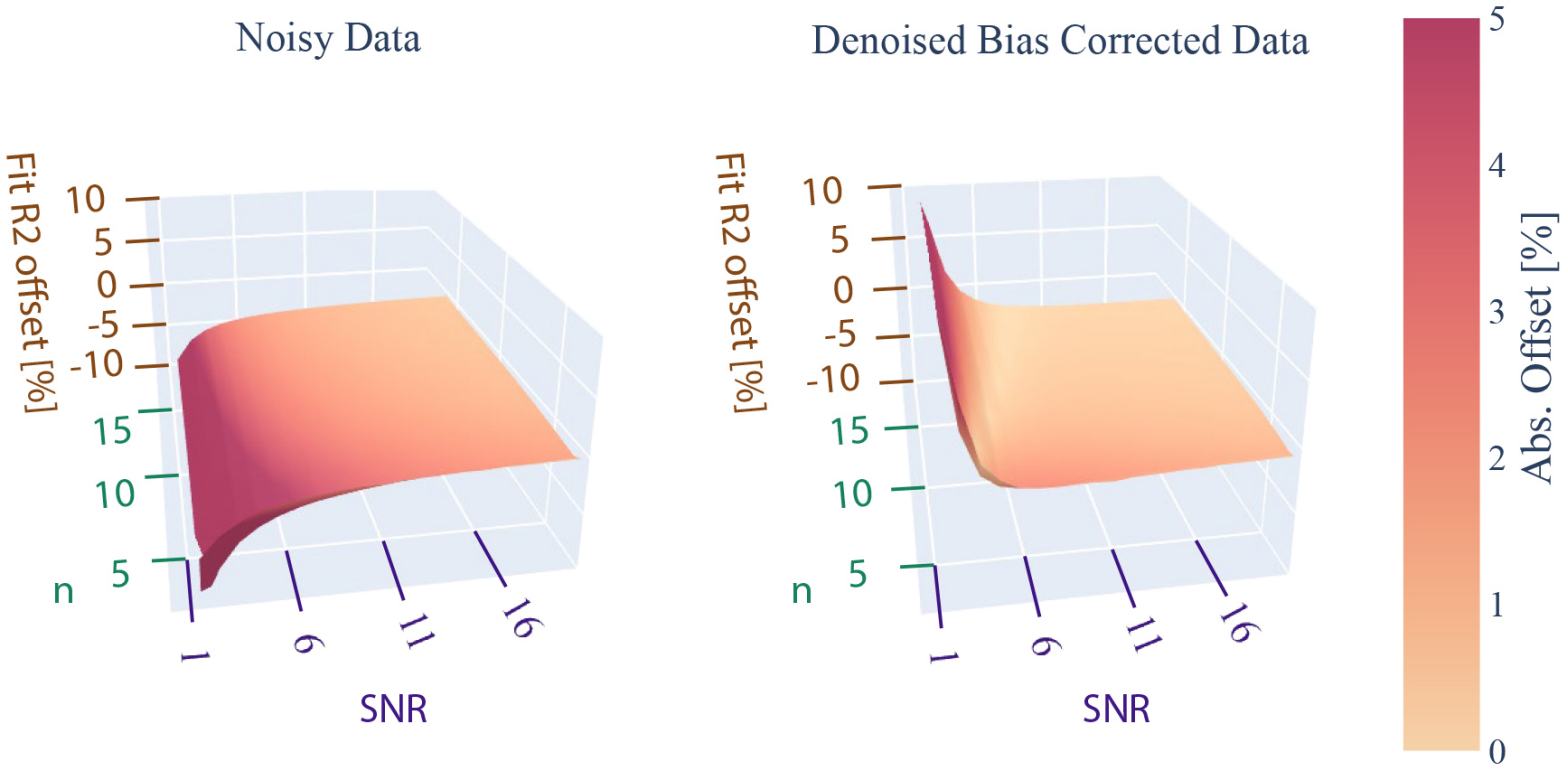
Bias in R_2_ estimates derived from 1000 noisy dictionary curves (left). Noise distributions were defined by SNR (see equation 16) and n parameters (see equation 13). After processing the curves with the denoising algorithm and applying magnitude bias correction, the absolute bias decreases (right). For easier visual comparison, the color coding uses the absolute value of the offset. A more homogeneous matching is achieved across the noise parameter space. However, at SNR approximately lower than 5, the bias correction is ineffective.

We processed the simulated noisy signal curves with the dictionary matching approach to estimate the ${\text{R}}_{2}$ value under the influence of noise with three different pre-processing types: (1) no pre-processing, (2) PCA denoising, (3) PCA denoising with magnitude bias removal. The pronounced effect on ${\text{R}}_{2}$ dictionary estimations is shown in Figure 3. The absolute fit mismatch is slightly decreasing toward higher SNR values. Inaccuracies are expected for low-SNR signal, as well as noise characteristics approaching pure Rician noise. PCA-based denoising does not substantially improve accuracy. Incorporating bias correction is necessary to improve fit accuracy (Fig. 3).

The influence of low SNR in high-resolution ${\text{R}}_{2}$ mapping is exemplified by the simulations. The best case scenario for the denoising performance is given using known noise characteristics for the bias removal, which is not necessarily the case for real data. Even in this optimal case, signals below an SNR of 5 could not be reliably corrected with the proposed method (i.e. irrespective of the accuracy of noise estimation). To mitigate the influence of those low-SNR voxel time series data, we excluded all voxels with SNR ≥5 from all further analyses. For the in vivo scans, this amounted to around 2−3.5% of the total voxel number, that is, 4−6% of brain voxels, measured after brain extraction. Affected voxels were solely found in the most inferior slices (see Fig. 9 for examples).

### 4.2 Quantitative ${\text{R}}_{\text{2}}$ estimation

#### 4.2.1 Phantom

**Fig. 4.**
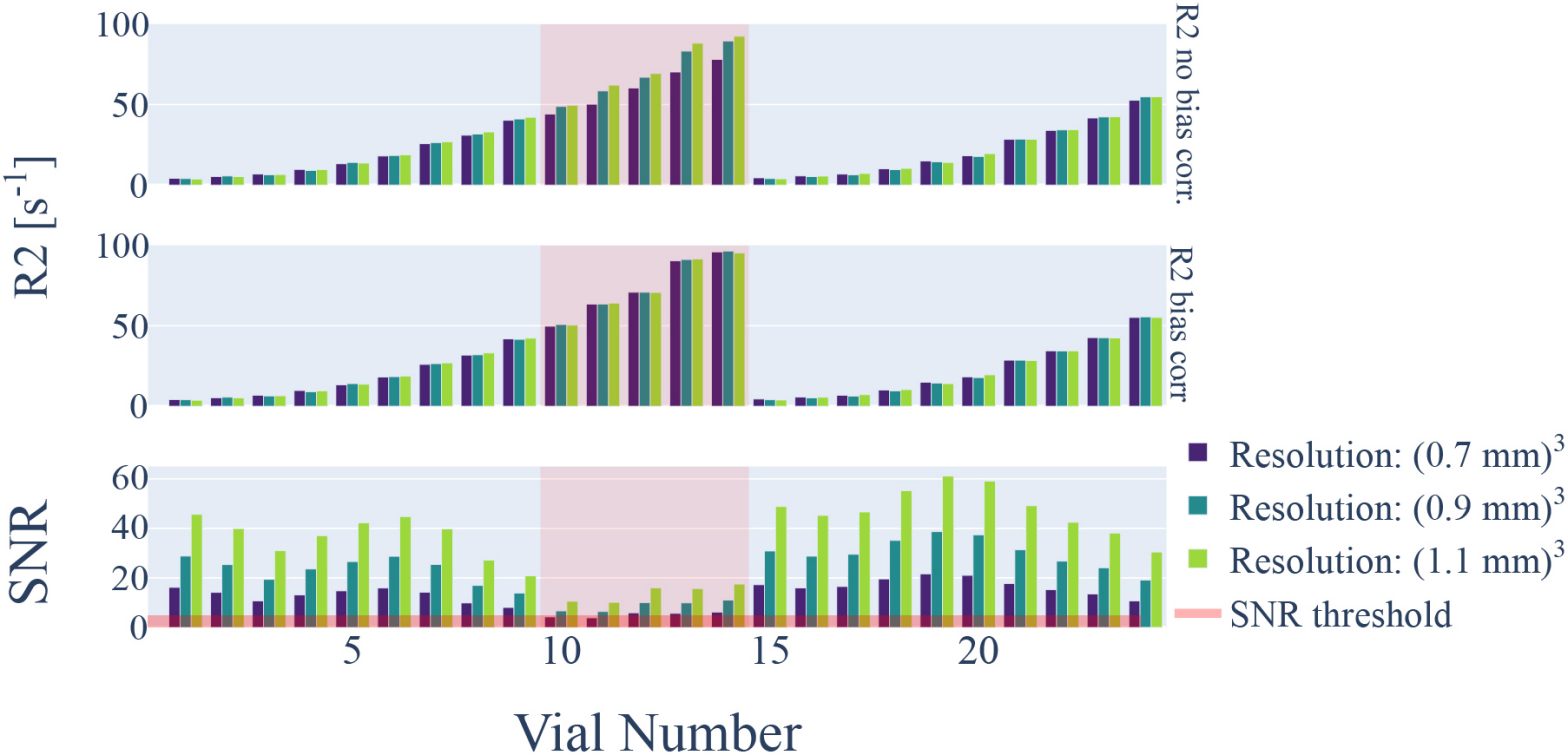
R_2_ estimation within a phantom containing vials with different ${\text{MnCl}}_{2}$ concentrations. The phantom was scanned at three different resolutions, from (0.7 mm)^3^ to (1.1 mm)^3^ isotropic resolution. The SNR is dependent on the vials position within the ${\text{B}}_{1}^{+}$/ $B_{1}^{-}$ profile but generally drops with decreasing voxel size. Low SNR leads to underestimation of R_2_ values (top row) if no bias correction is used. If bias correction is applied, ${\text{R}}_{2}$ estimation is stabilized and values remain comparable across resolution (middle row), respectively, SNR. The SNR is indicated in the bottom row. Vials with SNR ≤5 used to identify possibly poor estimates are highlighted by the red shaded area.

The bias due to low SNR predicted by the simulations was also observed in phantom measurements (Fig. 4). Different vials are subject to differences in SNR due to their position in the sample and the overall ${\text{B}}_{1}^{+}$ and $B_{1}^{-}$ profile. Additionally, the SNR scales with the used voxel sizes.

Higher resolution results in smaller voxels and consequently lower SNR. If the magnitude bias from noise was not accounted for, ${\text{R}}_{2}$ values decrease with lower SNR. ${\text{R}}_{2}$ bias of 10−20% was observed when the SNR dropped below 10, which is a realistic scenario for high resolution in vivo MESE measurements. Hence, bias correction is necessary to obtain quantitatively comparable R_2_ values across different resolutions.

**Fig. 5.**
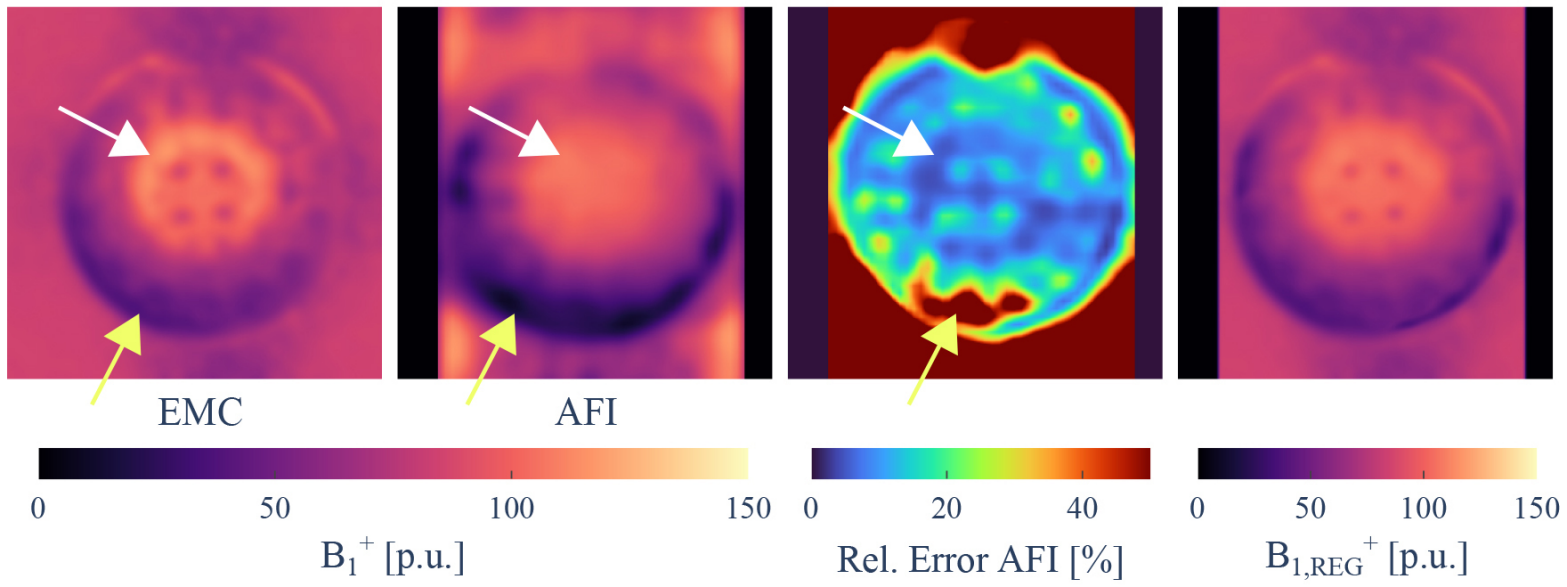
Estimated ${\text{B}}_{1}^{\;+}$ profile of (first column) EMC approach by simultaneous estimation of $\;{\text{T}}_{2}$ and ${\text{B}}_{1}^{\;+}$ without regularization in equation 17. For the AFI acquisition, the ${\text{B}}_{1}^{\;+}$ profile (second column) and the deduced relative error map (third column) are given. The estimated error is the highest for low ${\text{B}}_{1}^{\;+}\;$ areas (yellow arrows). For similarly large but positive deviations from 100% ${\text{B}}_{1}^{\;+}$ amplitude (white arrows), the relative error remains low. The ${\text{B}}_{1}^{\;+}$ estimates are combined using the AFI error map to produce the input for the regularization (fourth column).

The estimation of ${\text{B}}_{1}^{+}$ via the EMC method is compared with the estimate to the AFI acquisition in Figure 5. Although the EMC approach captures the overall ${\text{B}}_{1}^{+}$ transmit field bias qualitatively, substantial deviations from the AFI ${\text{B}}_{1}^{+}$ map were observed in the periphery and center of the phantom. As was shown at 4.7 T (McPhee & Wilman, 2016), our phantom measurements confirmed the limited capability of the EMC method to accurately estimate the ${\text{B}}_{1}^{+}$ profile. The combined estimation of ${\text{T}}_{2}$ and ${\text{B}}_{1}^{+}$ in equation 17 is an ill-posed problem without additional regularization.

However, the acquired AFI map only provides limited dynamical range and is prone to underestimation of ${\text{B}}_{1}^{+}$ in low ${\text{B}}_{1}^{+}$ areas (Yarnykh, 2010). Thus we used equation 11 to compute a ${\text{B}}_{1}^{+}\;$ map by weighted averaging of the EMC and AFI ${\text{B}}_{1}^{+}$ estimates. The weighting factor was based on the AFI error maps shown in Figure 5. We linearly down-weighted the contributions of the AFI ${\text{B}}_{1}^{+}$ estimate with increasing relative error estimate until a cutoff. The optimal threshold value ($\zeta_{\text{th}}=0.125$ ) was found by minimizing the coefficient of variation of the ${\text{R}}_{2}$ value within vials of identical ${\text{MnCl}}_{2}$ concentration. Unreliable estimation of the ${\text{B}}_{1}^{\;+}$ maps used for regularization resulted in increased ${\text{R}}_{2}$ variation across the vials with identical ${\text{MnCl}}_{2}$ concentration. The resulting ${\text{B}}_{1}^{\;+}$ maps are also shown in Figure 5. Further details about ${\text{B}}_{1}^{\;+}$ regularization and the influence of ${\text{B}}_{1}^{\;+}\;$ estimation on the variability within vials can be found in the Supplementary Material (S2).

_${\text{R}}_{2}$_ overestimation is observed in voxels attributed to low ${\text{B}}_{1}^{+}$ values (<50% ). The phantom study corroborates the previous simulation study that low ${\text{B}}_{1}^{+}$ significantly affects ${\text{R}}_{2}$ estimation. We deduce that this estimation bias can either be due to an inaccurate ${\text{B}}_{1}^{+}$ value upon regularization of the matching method, or be the result of low SNR, often correlating with a low ${\text{B}}_{1}^{+}$ profile.

**Fig. 6.**
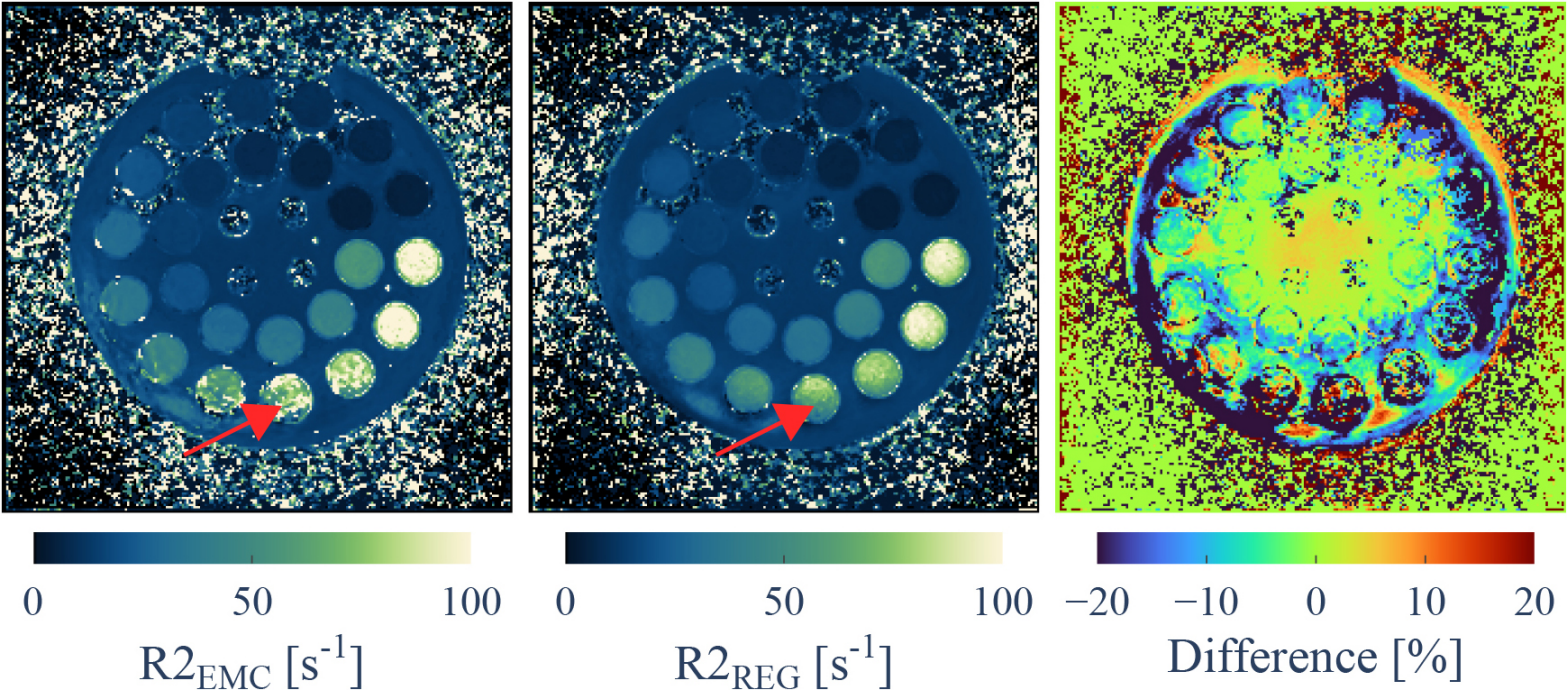
Estimated ${\text{R}}_{2}$ values for a single slice of a phantom MESE acquisition. Map generation was performed using no additional ${\text{B}}_{1}^{+}$ regularization (left) and with additional $\;{\text{B}}_{1}^{+}$ regularization (middle). The difference (no ${\text{B}}_{1}^{+}$ regularization minus ${\text{B}}_{1}^{+}$ regularization) is displayed (right). Residual bias in ${\text{R}}_{2}$ estimates (red arrows) is attributed to inaccurate $B_{1,\text{REG}}^{+}$ estimation and low SNR and observable especially in low ${\text{B}}_{1}^{+}$ areas.

_${\text{R}}_{2}$_ maps generated from the phantom MESE data using all corrections are shown in Figure 6. Regularization via $B_{1,\text{REG}}^{+}$ input stabilized the estimation, which is exemplified by comparison of vials containing similar ${\text{MnCl}}_{2}$ concentrations. Furthermore, the limits of ${\text{B}}_{1}^{+}$ correction via the EMC method only are apparent. The estimated ${\text{R}}_{2}$ values for the phantom vials are given in Table 1, containing additional estimates of ${\text{R}}_{2}$ obtained at 3 T and value references provided by the manufacturer. For 3 T, the values corresponded well to the reference provided by the manufacturer, considering neither errors in vial concentration nor value estimation was given by the vendor and vial manufacturing varies per batch. The concentration values were used to calculate the relaxivity for ${\text{MnCl}}_{2}$ to be $161.4{\text{ mM}}^{-1}{\text{s}}^{-1}$ , as previously reported for 7 T (Kellar & Foster, 1991).

**Table 1.** Estimated ${\text{R}}_{2}$ values for all voxels within vials of equal ${\text{MnCl}}_{2}$ concentrations.

_${\text{MnCl}}_{2}$_ [mM]	7 T ${\text{R}}_{2}$ [$s^{-1}$ ]	7 T ${\text{B}}_{1}^{+}$ [%]	3 T ${\text{R}}_{2}$ [$s^{-1}$ ]	vendor 3 T ${\text{R}}_{2}$ [$s^{-1}$ ]
0.017	4.49 ± 0.28	106.9 ± 12.3	2.08 ± 0.01	2.03
0.027	6.22 ± 0.35	103.8 ± 11.4	3.54 ± 0.26	3.05
0.039	8.47 ± 0.40	97.5 ± 19.2	5.49 ± 0.21	4.26
0.055	11.00 ± 0.36	109.2 ± 11.9	7.04 ± 0.30	5.88
0.078	14.81 ± 0.30	110.0 ± 8.0	8.89 ± 0.24	8.13
0.110	20.21 ± 0.45	98.9 ± 12.7	11.66 ± 0.36	11.11
0.159	28.72 ± 0.69	86.1 ± 14.8	15.80 ± 0.52	16.67
0.194	34.84 ± 0.95	97.8 ± 19.2	18.55 ± 0.59	20.0
0.245	43.53 ± 1.77	79.0 ± 26.8	22.37 ± 0.86	25.0
0.320	54.01 ± 5.67	80.1 ± 33.4	27.69 ± 1.32	33.33
0.480	75.49 ± 9.94	55.3 ± 5.2	37.49 ± 2.42	50.0
0.630	97.49 ± 5.37	85.37 ± 7.5	46.24 ± 2.81	66.67

The standard deviation appeared to be influenced by the $B_{1}^{+}$ value. That is, lower $B_{1}^{+}$ values or a high $B_{1}^{+}$ variation translated into higher variation within the $R_{2}$ estimates. For $B_{1}^{+}$ values approaching 50%, the accuracy in $R_{2}$ estimation was considered low.

#### 4.2.2 In vivo brain imaging

##### 4.2.2.1 Pre-processing

**Fig. 7.**
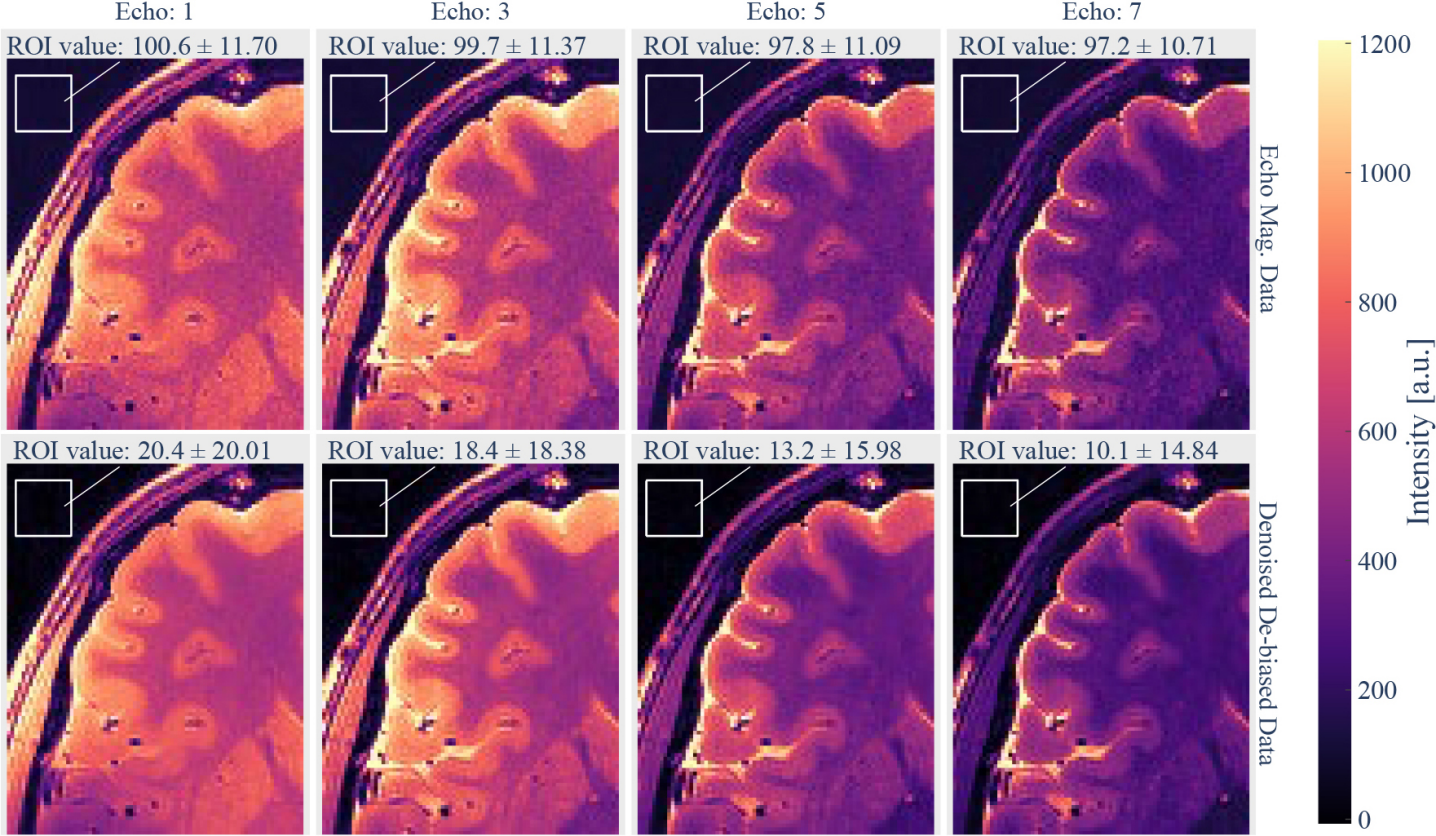
Example of denoising and bias correction efficiency in vivo. The same section within a slice of MESE echo magnitude data is shown for different echoes. In the top row, images show grainy noise, with a nearly constant mean magnitude value observed in voxels outside the head. After PCA denoising and bias correction (bottom row), the bias and image noise are reduced. We indicate an average ROI value in the image background to highlight the noise bias removal. However, the bias removal is used on all image voxels.

For the in vivo data, the denoising and bias correction approach increased data quality and consistency (Fig. 7). The noise floor was substantially reduced by the bias correction, especially in areas outside the head used for noise estimation.

##### 4.2.2.2 ${\text{R}}_{\text{2}}$ estimation

The data of the participant scanned twice in different sessions were subjected to the whole processing pipeline. For scan–rescan reproducibility evaluation, the ${\text{R}}_{2}$ estimates of the ROIs of both scans were taken per voxel. A Pearson correlation coefficient calculation between both measurements was r(507)=0.9875 for the 507 measurement pairs. The Bland–Altman plot and further details for the repeated ${\text{R}}_{2}$ measurements for the ROIs can be found in the Supplementary Material (S3). We found value-dependent deviation toward lower ${\text{R}}_{2}$ ROI mean values resulting in a higher spread around the mean value. It could be shown that the variation of ${\text{R}}_{2}$ over different scan sessions was well within the inter-subject variability given in Table 2. Since the latest echo was sampled at 63 ms , this might hint at a reduced measurement accuracy toward low-rate changes in the signal. The coefficient of variation was found to be 3.62% .

**Table 2.** ${\text{R}}_{2}$ values for different cortical and subcortical brain areas, as well as overall white matter structure.

Label	Estimated ${\text{R}}_{2}$ [$s^{-1}$ ]	Literature ${\text{R}}_{2}$ [$s^{-1}$ ]
Cortex - temporal lobe	20.42 ± 3.35	
Cortex - parietal lobe	22.93 ± 3.21	
Cortex - frontal lobe	21.85 ± 3.10	
Cortex - occipital lobe	22.96 ± 3.57	
Cortex - insula	20.43 ± 2.37	
Cingulate cortex	21.71 ± 2.81	_∼_ 23 [2]
Average GM	21.87 ± 3.36	18.18 ± 0.66 [1]
White matter	24.75 ± 3.08	25.64 ± 3.29 [1], ∼ 21.3 [2]
Putamen	28.16 ± 3.89	_∼_ 26 [2], 30 [3]
Pallidum	39.96 ± 2.76	_∼_ 43 [3]
Thalamus	25.62 ± 4.15	24.39 ± 2.38 [1], ∼ 24 [2]
Caudate	25.11 ± 3.76	_∼_ 28 [3]
Brain stem	21.50 ± 3.57	_∼_ 19 [3]
Substantia nigra	38.12 ± 6.58	_∼_ 42 [3]
Nucleus accumbens	21.72 ± 2.56	_∼_ 27 [3]
Hippocampus	19.35 ± 2.76	_∼_ 18 [3]
Nucleus ruber	33.49 ± 4.26	_∼_ 34 [3]

Literature values are taken from [1] Emmerich et al. (2018), [2] Wiggermann et al. (2020), and [3] van Gelderen et al. (2024).

**Fig. 8.**
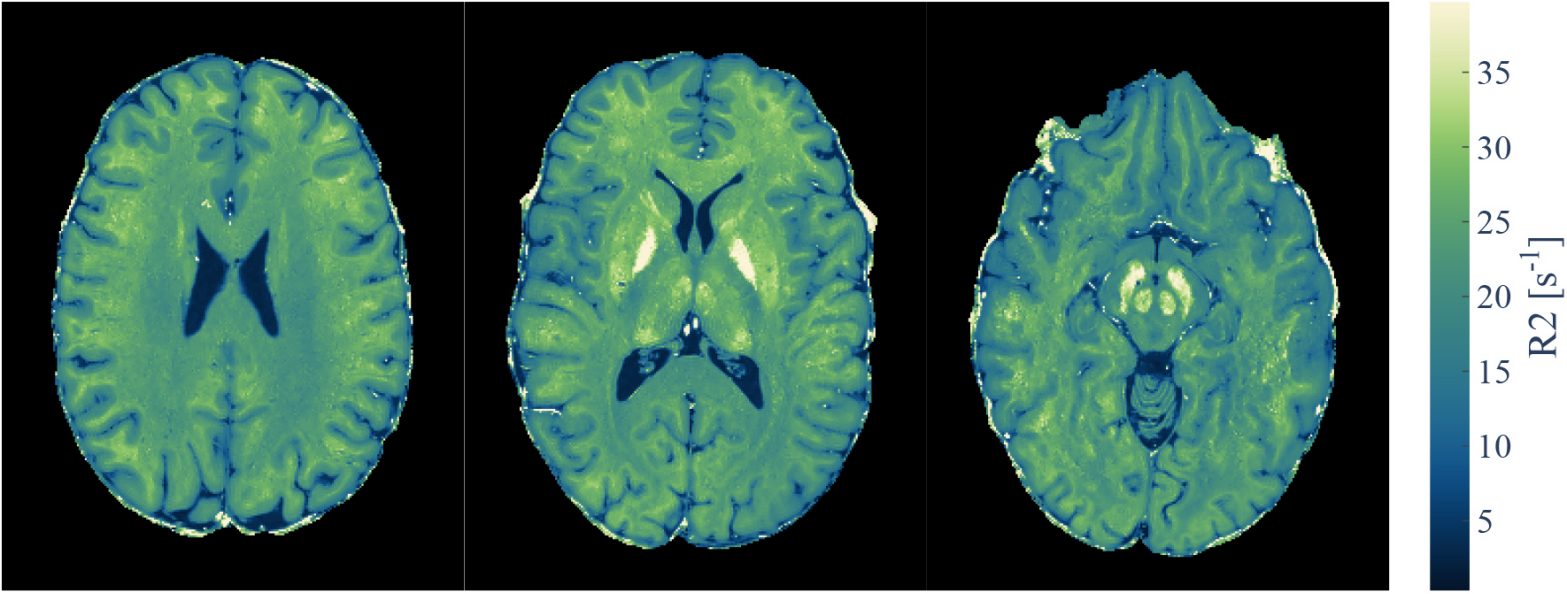
R_2_ maps for three axial slices from a single participant generated from an in vivo MESE acquisition acquired with ${\left(0.7\text{ mm}\right)}^{3}$ isotropic resolution.

Exemplary three slices of an ${\text{R}}_{2}$ map of one participant are shown in Figure 8. Gray matter/white matter (WM) contrast is most pronounced in frontal brain areas and decreased in occipital areas.

_${\text{R}}_{2}$_ variations in WM might be caused by orientation of fiber bundles or underlying tissue differences. Iron-loaded subcortical areas such as the substantia nigra and nucleus ruber were well delineated. Low ${\text{B}}_{1}^{+}$ values in inferior slices resulted in underestimation of ${\text{R}}_{2}$.

**Fig. 9.**
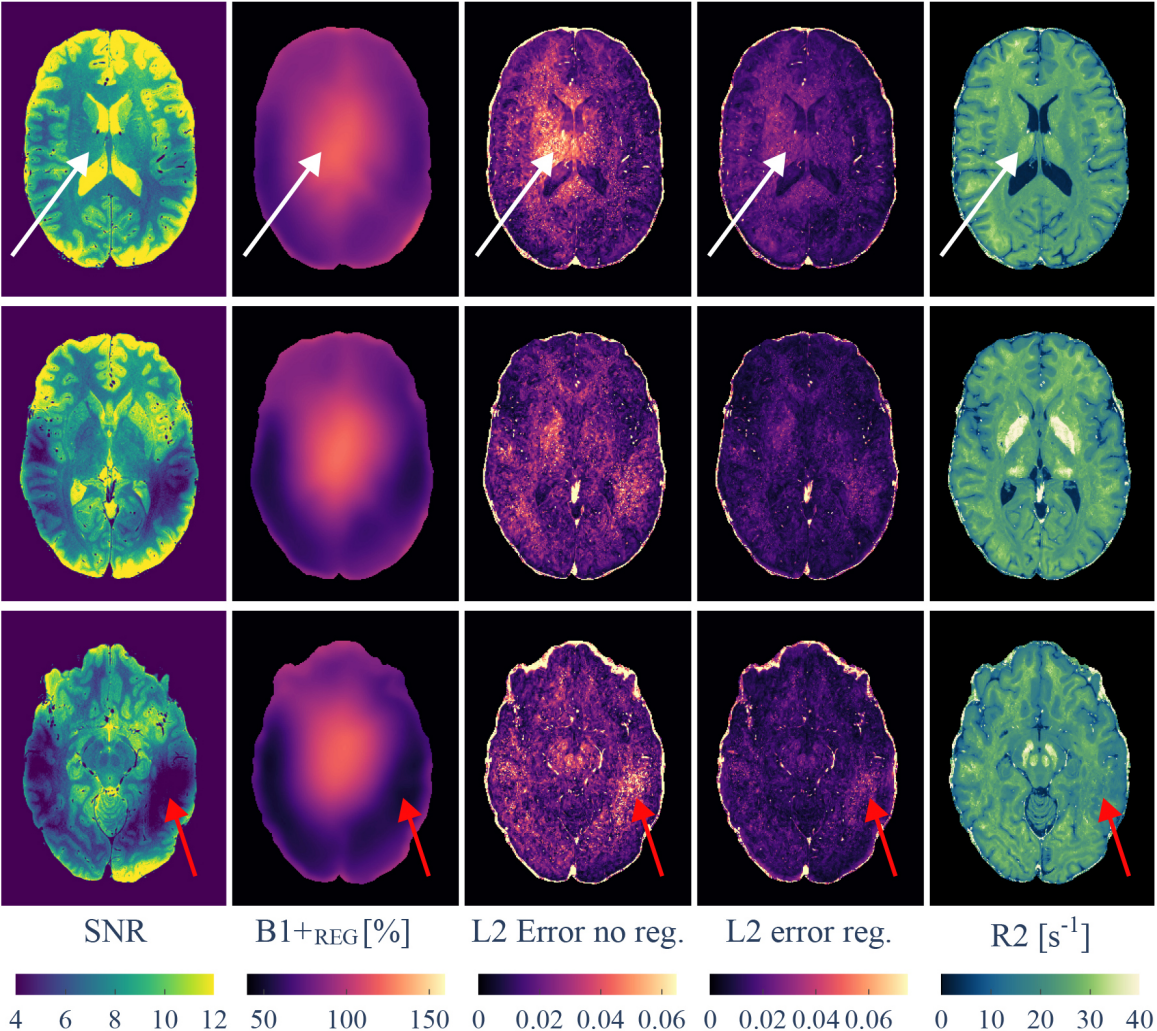
Three slices of MESE and AFI data from a single participant. After noise estimation, an SNR map is computed for the MESE data (first column) using equation 16. ${\text{B}}_{1}^{+}$ estimates from AFI (second column) correlate with SNR: low ${\text{B}}_{1}^{+}$ yields low voxel SNR (red arrows). ${\text{B}}_{1}^{+}$ varies considerably at 7 T. The dictionary L2 minimization error (third column; first term in equation 17) also correlates with ${\text{B}}_{1}^{+}$, with larger errors in areas of transmit field deviation. The total error with ${\text{B}}_{1}^{+}$ regularization (fourth column) is reduced in regions of high ${\text{B}}_{1}^{+}$ (white arrows), helping to avoid ${\text{R}}_{2}$ underestimation. In low-SNR regions (red arrows), both errors are high, indicating failed dictionary matches. Estimated ${\text{R}}_{2}$ maps (fifth column) show high contrast and robustness to ${\text{B}}_{1}^{+}$ variation, except where SNR is very low.

Especially toward inferior brain areas, most prominently in the temporal lobes, ${\text{B}}_{1}^{+}$ can drop substantially at 7 T (Fig. 9). The proposed method was not able to satisfactorily recover the induced bias in these areas. However, for SNR values ≥5 , the method was largely insensitive to SNR or ${\text{B}}_{1}^{+}$ variations.

**Fig. 10.**
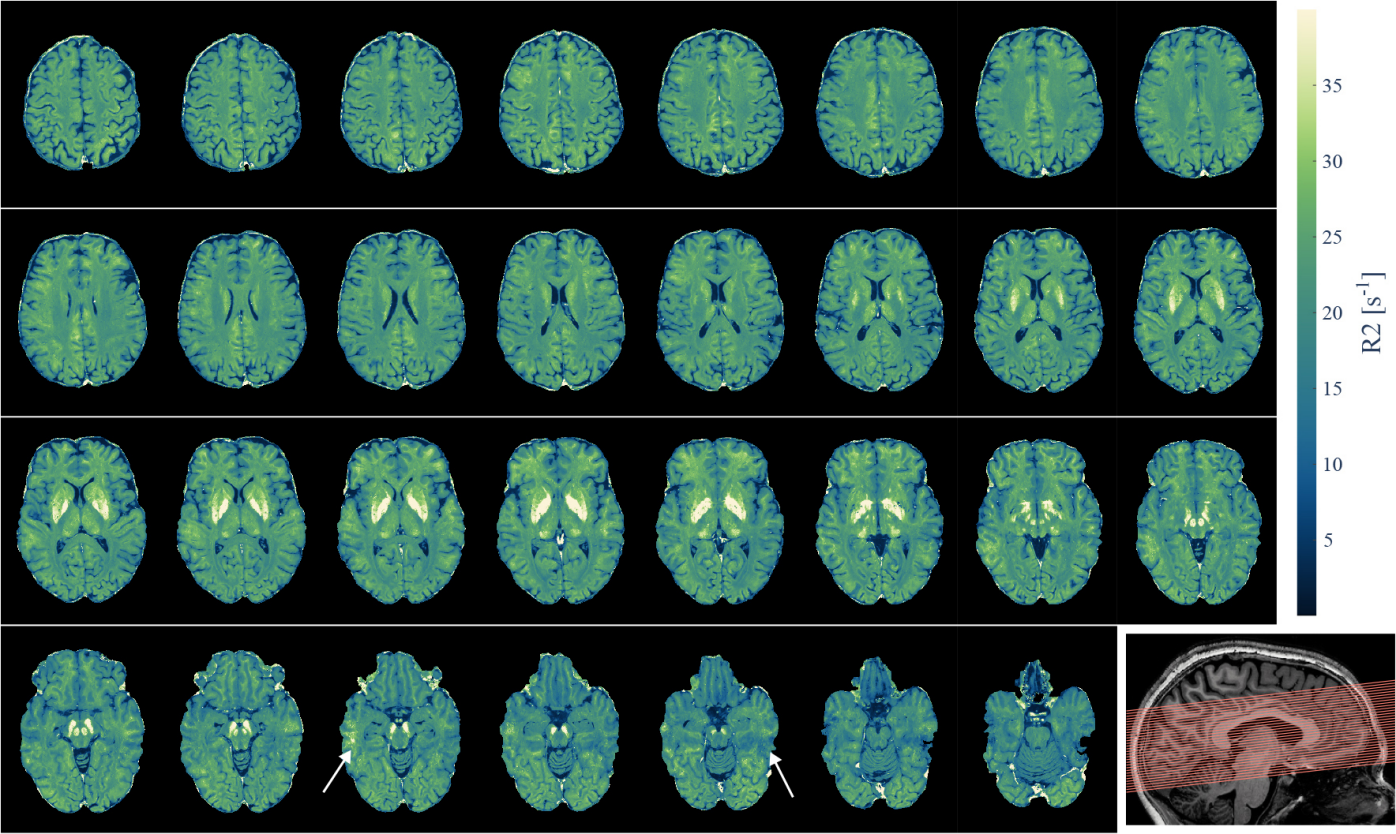
${\text{R}}_{2}$ map of a single participant. High-resolution ${\text{R}}_{2}$ estimation is feasible across various brain regions. Toward inferior slices, the low ${\text{B}}_{1}^{+}$ field and/or low SNR at 7 T introduced bias which was not fully mitigated (e.g., white arrows). Iron-rich subcortical structures (e.g., basal ganglia, nucleus ruber, substantia nigra) were clearly visible as hyperintense structures. Note: The slices are placed with a signal-free inter-slice gap, see bottom right image for reference of slice placement.

Figure 10 shows all slices of an ${\text{R}}_{2}$ map generated for a different participant. The estimated ${\text{R}}_{2}\;$ values for all ROIs are given in Table 2. ${\text{R}}_{2}$ values were within the range of literature findings where available (Emmerich et al., 2018; Schmidt et al., 2021; van Gelderen et al., 2024; Zhu et al., 2014). However, GM mean values were higher than the results obtained by Emmerich et al. (2018) for similar age groups, who reported for GM $R_{2}=18.18\pm 0.66\;s^{-1}$ , for WM $R_{2}=25.64\pm 3.29\;s^{-1},$ and for thalamus $R_{2}=24.39\pm 2.38\;s^{-1}$ . Additionally, within GM structures, regional differences were found (Table 2). Fly-through animations of the estimated ${\text{R}}_{2}$ maps for all five participants can be found in the Supplementary Material (S4).

**Fig. 11.**
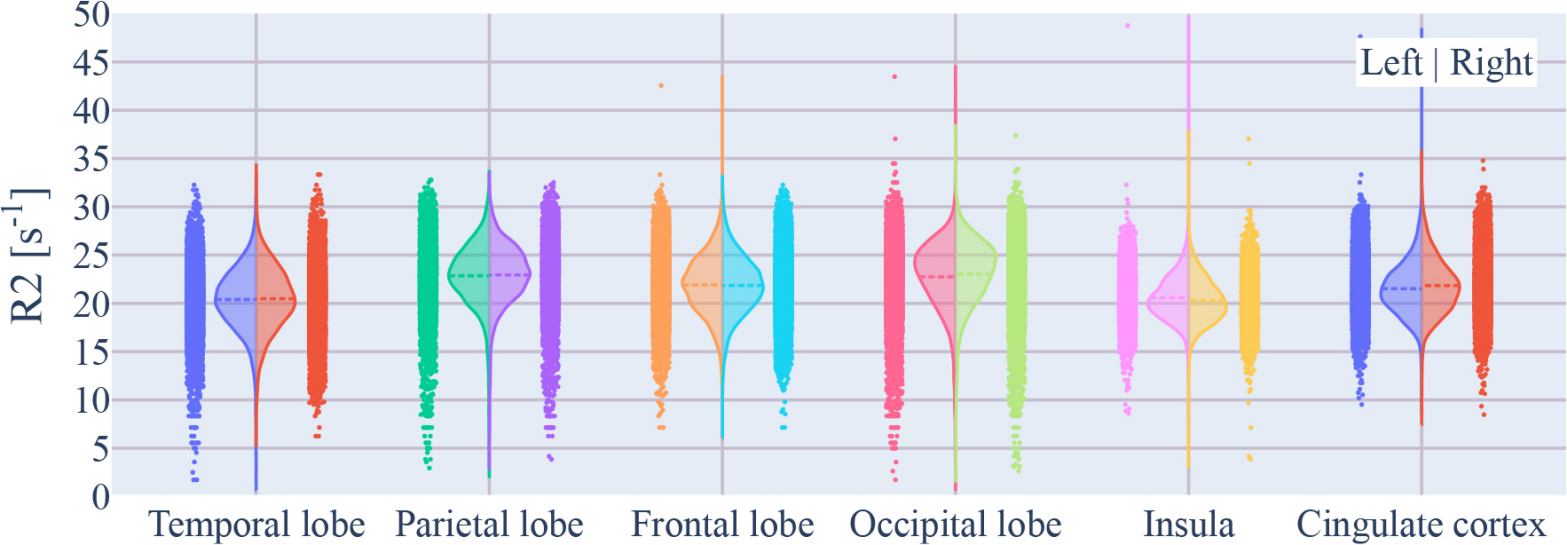
${\text{R}}_{2}$ value distributions pooled across all voxels and participants within each cortical ROI.

Figure 11 shows the distribution of ${\text{R}}_{2}$ values for all GM regions for both hemispheres. Parietal and occipital areas showed higher ${\text{R}}_{2}$ mean values than other brain regions. The values might reflect a gradient in cortical myelination as previously reported (Huntenburg et al., 2018).

The ${\text{R}}_{2}$ sampling from the middle cortical band was primarily used to decrease potential partial volume effects, for example, close to neighboring CSF voxels that show very low ${\text{R}}_{2}$ values. However, as we only sample from the cortical midline, we were insensitive to ${\text{R}}_{2}$ variations across the cortical depth caused by differences in myeloarchitecture.

**Fig. 12.**
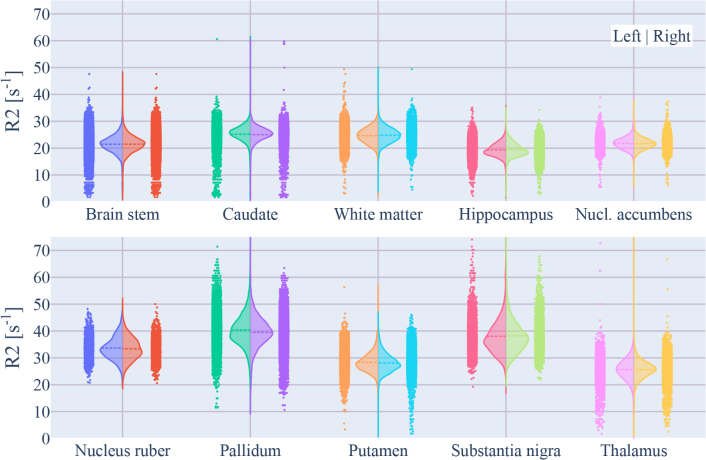
Distribution of ${\text{R}}_{2}$ values for sub-cortical and white matter ROI voxels pooled across all participants. For the brain stem, only the overall distribution pooled across both sides was plotted. Otherwise, distributions for all ROIs are presented separately for the left and right hemispheres.

Interesting value differences can further be found for subcortical structures in Table 2. Subcortical and WM value distributions are shown in Figure 12. Most prominently, pallidum, substantia nigra, and nucleus ruber were well visible due to high ${\text{R}}_{2}$ values, related to their high iron content (Hallgren & Sourander, 1958; Langkammer et al., 2010). Average WM structures showed slightly higher ${\text{R}}_{2}$ values than GM regions. However, ${\text{R}}_{2}$ of WM is not constant throughout the brain. Possible dependence of ${\text{R}}_{2}$ on fiber bundle orientations as well as different myelination levels will affect the quantitative value.

## 5 Discussion

We successfully obtained high-resolution ${\text{R}}_{2}$ maps at 7 T using a modified MESE product sequence in combination with a ${\text{B}}_{1}^{+}$ regularized dictionary matching approach. The effect of ${\text{B}}_{1}^{+}$ and RF slice profile on the signal was modeled using Bloch equation simulations. Reliable estimates of ${\text{R}}_{2}\;$ were obtained for most of the brain, except for some inferior regions of the brain. The ${\text{R}}_{2}$ maps allowed for delineating iron-rich subcortical structures and reflected region-specific variations, which might be attributed to, among others, variations in cortical myelination or vascular structure. It was shown via simulations and phantom experiments how low SNR affects ${\text{R}}_{2}$ mapping by the introduction of a non-zero signal amplitude asymptote. Denoising and magnitude bias correction before dictionary fitting effectively reduced the bias in ${\text{R}}_{2}$ values and improved the quality of ${\text{R}}_{2}$ maps.

We determined mean ${\text{R}}_{2}$ values in ROIs encompassing multiple cortical and subcortical structures, including the nucleus ruber, substantia nigra, and pallidum, which appeared hyperintense on ${\text{R}}_{2}$ maps due to their high iron content. For cortical ROIs, we extracted mid-layer cortical ${\text{R}}_{2}$ values, avoiding partial voluming with white matter and/or CSF.

Quantitatively, our ${\text{R}}_{2}$ values aligned with previous findings (Emmerich et al., 2018; Schmidt et al., 2021; Wiggermann et al., 2020; Zhu et al., 2014). However, Wiggermann et al. (2020) reported lower geometric mean ${\text{R}}_{2}$ values in WM, Emmerich et al. (2018) reported lower ${\text{R}}_{2}$ values especially in GM structures. These discrepancies could be due to differences in ROI placements or acquisition protocols, such as partial volume effects, longer echo spacing, and k-space signal mixing between echoes in turbo-spin echo sequences (Busse et al., 2008). Another factor could be the correspondence of resolution-dependent noise influence on the dictionary matching which has previously not been accounted for. We also observed a trend of higher ${\text{R}}_{2}$ values in occipital and parietal cortical areas, consistent with findings from previous studies (Huntenburg et al., 2018). Qualitatively, this resulted in less pronounced GM/WM contrast in the ${\text{R}}_{2}$ maps of these brain areas.

The accuracy of the dictionary-based fitting method depends on noise characteristics, influenced by details of the RF receive chain and image reconstruction. The acquired data were processed using a PCA-based filter denoising method that noticeably increased the image quality. Patch dimensions of the PCA method and the chosen fixed singular value cutoff threshold were mainly based on the number of available echoes and visual inspection of the performance. Due to the low number of echo images and thus patch dimensionality, the choice for the threshold is limited. The use of patches built using similarity measures between voxel signal (Li et al., 2024) or patch grouping based on previous segmentation might further improve the performance. In future work, denoising ideally should be done at an earlier stage in the image reconstruction pipeline, as subsequent interpolation steps will alter the noise characteristics (Bazin et al., 2019).

The magnitude noise bias varies with the experimental setup’s noise characteristics and used imaging coils (Dietrich et al., 2008). To achieve robust ${\text{R}}_{2}$ values, a noise bias correction approach is necessary and its performance depends on an accurate noise estimation. It has to be mentioned that, in our model, noise was assumed to be stationary in an image slice. However, spatial variation of noise characteristics can be significant, dependent on coil sensitivity profiles and the reconstruction mechanisms used (Aja-Fernández & Vegas-Sánchez-Ferrero, 2016). Informing the noise estimation with details about the reconstruction, if available, may further improve ${\text{R}}_{2}$ map quality.

As expected at 7 T, accounting for the imperfect transmit field ${\text{B}}_{1}^{+}$ is crucial for precise ${\text{R}}_{2}$ estimation. ${\text{B}}_{1}^{+}$ inhomogeneities are exacerbated (Van de Moortele et al., 2005) at higher fields and thereby diminish the efficacy of the EMC technique due to its poor performance in simultaneously estimating ${\text{R}}_{2}\;$ and ${\text{B}}_{1}^{+}$ (McPhee & Wilman, 2016). To address this challenge, we incorporated an AFI ${\text{B}}_{1}^{+}$ reference map. However, the AFI ${\text{B}}_{1}^{+}$ mapping method was shown to have limited dynamic range (Yarnykh, 2010). As a result, a residual error in ${\text{R}}_{2}$ estimation might still be present and originate from imprecise ${\text{B}}_{1}^{+}$ estimates, especially for regions with higher ${\text{B}}_{1}^{+}$ deviations. Achieving a more homogeneous ${\text{B}}_{1}^{+}$ field is possible by the use of targeted ${\text{B}}_{1}^{+}$ shimming either across the whole brain or within specific ROIs (Kazemivalipour et al., 2023; Padormo et al., 2015). Recent research also suggests that the application of parallel transmit techniques such as the use of universal RF pTx pulses (Deniz, 2019) could potentially homogenize the ${\text{B}}_{1}^{+}$ profile to an extent, which can be processed as shown by our study. Overall, minimizing the ${\text{B}}_{1}^{+}$ inhomogeneities is crucial for enhancing the accuracy of any ${\text{R}}_{2}$ mapping technique. This is especially true for regions with low inherent voxel SNR, exacerbated by the small voxel sizes in high-resolution imaging. These improvements may enable access to brain areas with very low SNR, such as the inferior temporal lobes at 7 T.

Our study also reports ${\text{R}}_{2}$ values of white matter where the limitations of mono-exponential signal decay models were clearly demonstrated and affect simple single ${\text{R}}_{2}$ estimates (A. MacKay et al., 2006). We did not focus on specific substructures and orientation dependencies of the underlying tissue which may result in a mixture of multiple relaxation compartments. Our method does not account for multi-exponential ${\text{R}}_{2}$ decay, which would require a denser sampling of echoes. Acquiring more echoes by repetitive use of refocusing pulses increases both the SAR of the sequence and the scan time due to longer echo train length. Applying a mixed Gaussian model to dictionary curves in the fitting process may enable multi-compartmental ${\text{R}}_{2}\;$ estimation, but the needed number of echoes for such modeling is difficult to estimate a priori. Extending the method to include multiple components with different ${\text{R}}_{2}$ characteristics using auto-encoder AI architectures might be feasible, as forward modeling facilitates training data creation (Doucette et al., 2021). Similarly, the short echo train duration is beneficial to capture GM and WM tissue ${\text{R}}_{2}\;$ but might not be sufficient to map low ${\text{R}}_{2}\;$ components such as CSF accurately. Achieving this with the limited number of echoes per curve remains a goal for future work.

An advantage of our approach is the relatively short scan time of 10 :12 min required to obtain sub-millimeter ${\text{R}}_{2}$ maps in vivo. However, overall coverage is still limited. Thus, the method is ideal for high-resolution studies of targeted ROIs. Achieving whole brain coverage requires compromises in resolution and/or scan time. Additional gradient echo sampling might increase data sampling while reducing the number of RF pulses, allowing for lower inter-echo train time and consequently the scanning of more slices within one $T_{R}$. However, the echo spacing in such approaches might increase to accommodate more readouts, preventing fast ${\text{R}}_{2}$ decay estimation. In the MESE sequence, the acquisition of more slices results in longer $T_{R}$ and increased scan times. Longer scan times may require motion correction techniques for robust acquisitions (Vaculčiaková et al., 2022). Particularly in clinical settings, maintaining feasible scan times while achieving whole brain coverage is crucial. Optimizing MR data acquisition and reconstruction, deviating from standard vendor sequences, can achieve this (Schmidt et al., 2023). Recent reconstruction methods such as J-LORAKS (Bilgic et al., 2018; Haldar, 2014) could leverage some of the spatial redundancies available in time series acquisitions and enable further acceleration. However, both J-LORAKS and sequence design require additional software and are challenging to implement on conventional scanning platforms.

## 6 Conclusion

High-resolution ${\text{R}}_{2}$ mapping facilitates investigation into the brain’s microstructure, modeling of contrast mechanisms and studies of the healthy and pathological brain. Our study presents a method for ${\text{R}}_{2}$ mapping in the human brain at 7 T. ${\text{R}}_{2}$ could be estimated by Bloch simulation-based dictionary fitting for the majority of the brain, except for some inferior brain areas with low ${\text{B}}_{1}^{+}$ fields and SNR. A denoising pre-processing step incorporating magnitude bias correction effectively reduced image noise and produced accurate ${\text{R}}_{2}$ values. Human brain ${\text{R}}_{2}\;$ estimates were in line with the literature, reflecting variations in cortical myeloarchitecture and highlighting iron-rich subcortical structures. Remaining bias in the presented method is due to high ${\text{B}}_{1}^{+}$ inhomogeneity and further research is needed to reduce ${\text{B}}_{1}^{+}$ inhomogeneities to a correctable level across the entire brain.

## Supplementary Material

Supplementary Material

## Data Availability

The software used in this study is available on GitHub at https://github.com/schmidt-jo/PyMRItools (Schmidt & Scheibe, 2024). This includes the EMC method, which includes dictionary generation and signal-matching reconstruction methods, the implementation of the PCA-based denoising and noise bias correction, the Gibbs ringing artifact removal algorithm, and a mono-exponential fitting algorithm. The package was implemented in Python using PyTorch for accelerated GPU calculations. The code repository contains phantom data that allows for reproducing the experiments. The presented subject data contain sensitive patient information and is subject to European GDPR compliance. It is thus only available upon request and with a formal data sharing agreement.
