# Supplementary material for: High-resolution quantitative T2 mapping of the human brain at 7 T using a multi-echo spin-echo sequence and dictionary-based modeling
Source: Imaging Neurosci (Camb). 2025 Jul 25;3:IMAG.a.81. doi: 10.1162/IMAG.a.81 (PMC12330843; doi:10.1162/IMAG.a.81)
Supplement: Supplementary Material [file IMAG.a.81_supp.pdf]

Supplementary Materials

S1 Phantom

The labels and positions of all vials are given in Figure S1. Respective phantom vial concentrations are detailed in Table S1.

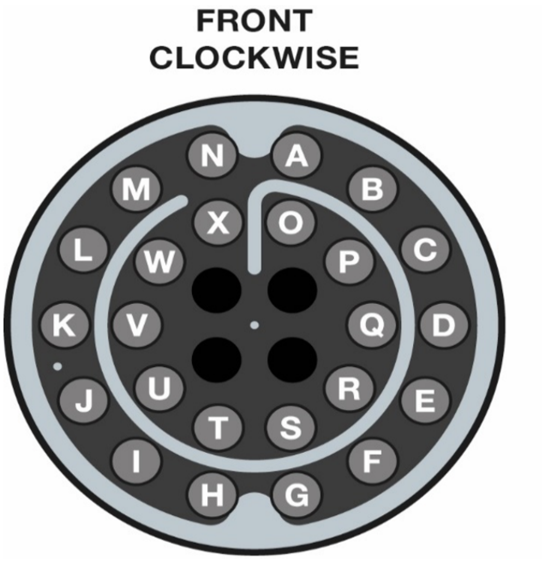

Figure S1: Phantom vial labels and positions in the cylindrical phantom.

Loc.	$MnCl_2$ [mM]	—	Loc.	$MnCl_2$ [mM]
A	0.017		M	0.630
B	0.027		N	0.630
C	0.039		O	0.017
D	0.055		P	0.027
E	0.078		Q	0.039
F	0.110		R	0.055
G	0.159		S	0.078
H	0.194		T	0.110
I	0.245		U	0.159
J	0.320		V	0.194
K	0.480		W	0.245
L	0.480		X	0.320

Table S1: Nominal $MnCl_2$ concentrations per vial.

S2 B_1^+ regularization map creation

The corresponding combined regularization map was calculated for a number of threshold values and used as input for the regularization. The optimal threshold value was then found by minimizing the coefficient of variation of the R_2 value within vials of identical $MnCl_2$ concentration.

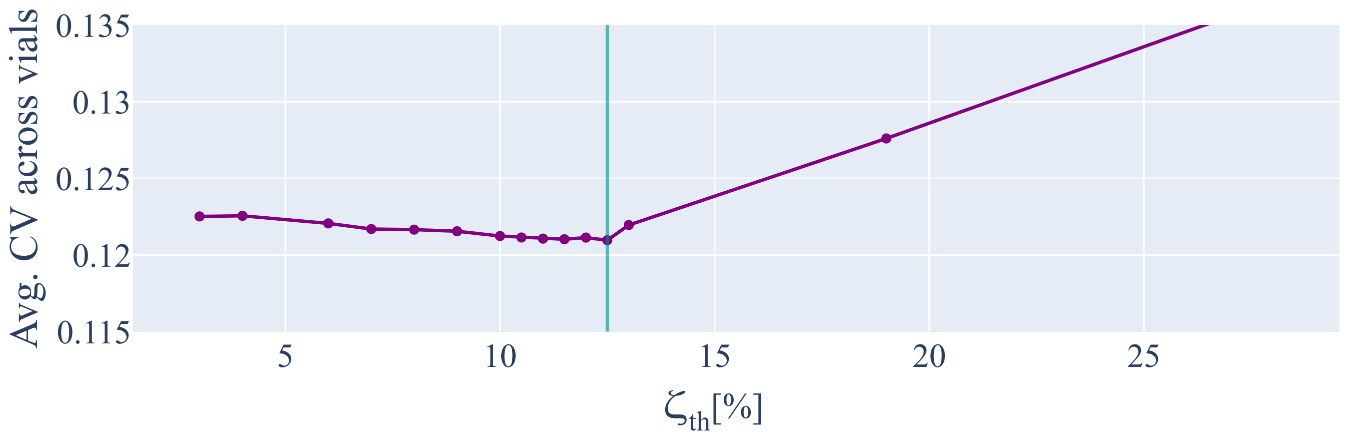

Figure S2: The average coefficient of variation within vials of identical $MnCl_2$ concentration is plotted against the weighting threshold of the AFI B_1^+ map. A minimum of R_2 vial variation can be found for an error threshold of 12.5%, which roughly corresponds to AFI B_1^+ estimates below 50%.

The influence of B_1^+ on R_2 value estimation of identical $MnCl_2$ values can be seen in Figure S3. Since each concentration was present in two vials, voxels from different vials with same concentrations were affected differently by B_1^+.

It can be observed that low B_1^+ efficiency causes overestimation of R_2 values. In turn, moderate B_1^+ mismatch yielded narrow and uniform R_2 distributions across vials. However, significant B_1^+ variability led to a spread in the distribution or bimodal R_2 distributions, where lower B_1^+ correlates with higher R_2 values. This underlines the influence of the B_1^+ estimation for R_2 estimation using EMC dictionary matching at 7 T.

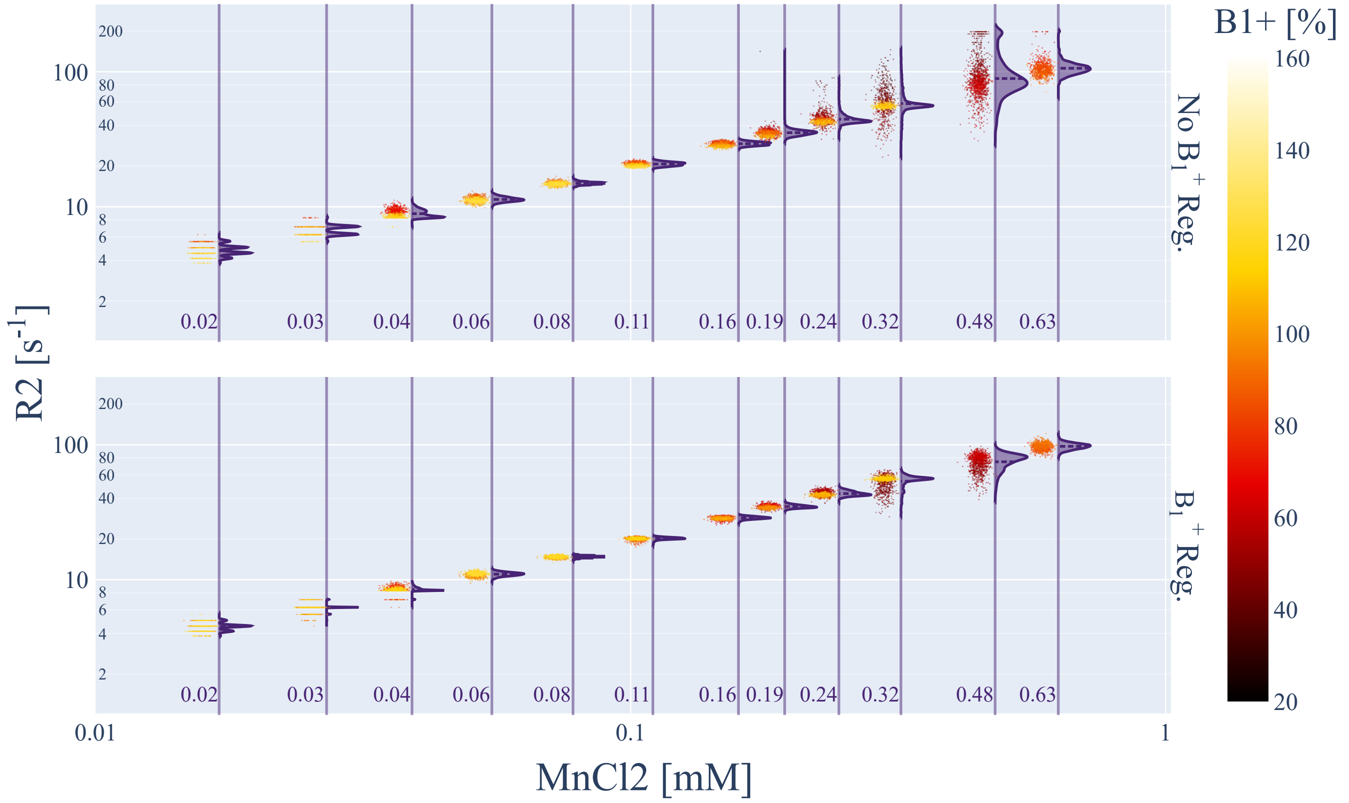

Figure S3: The R_2 estimates for a number of datapoints from a phantom MESE acquisition is plotted against the MnCl_2 concentration in the vials. Given are values estimated without (top row) and with (bottom row) B_1^+ regularization upon dictionary matching. Mean and distribution characteristics for each concentration are shown. Datapoints are color-coded with the color indicating B_1^+ offset as estimated by the B_1^+ map. It can be seen that the approach yielded narrow R_2 estimate distributions when the B_1^+ value remained close to the optimal value. In turn, when B_1^+ was low, accuracy was reduced resulting in overestimation of R_2 as well as a broad value distribution. The B_1^+ regularization reduced the overall width of the distributions. Any discretization in the R_2 value is due to the chosen simulated dictionary parameters, which were chosen to be sparse for lower R_2 values.

S3 Scan - Rescan Testing

Since the evaluated ROIs have different sizes we randomly drew at most 20 equal sized subsets from each ROI. The subset size was given by the smallest ROI and the number of draws by the size relation of the respective ROI to the smallest. This way we collected 507 draws from the 16 ROIs, however, the ROIs are not represented equally in the analysis. We evaluated the mean R_2 for all of those subsets. A Pearson correlation coefficient calculation between both measurements was $r(507) = 0.9875$ for the 507 measurement pairs. The plot is given in Figure S4 for R_2 estimation without and with noise bias correction. Both comparisons show a shift of 5.65% and 1.21%, respectively, between the R_2 difference means. Whereas the noise processed data shows a slight shift in the mean R_2 values for higher R_2

estimates as well as a lower spread in the relative difference.

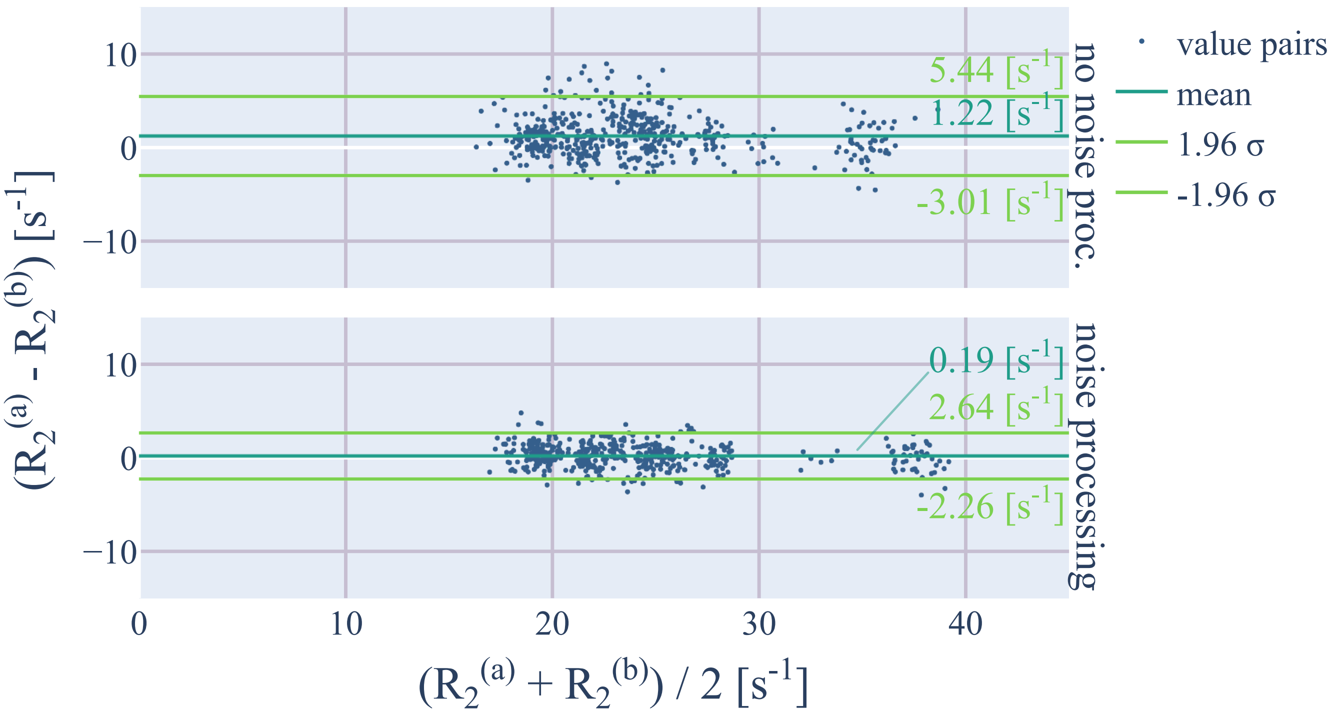

Figure S4: Bland-Altman plot of the R_2 values of the used ROIs from scanning of the same subject in 2 different sessions (a and b). The first row shows the R_2 estimates when no noise bias correction was applied. A shift of $1.22[\mathrm{s}^{-1}]\%$ in the mean difference was visible. The coefficient of variation was 7.35%. Comparing the noise bias corrected R_2 estimates (second row), the spread in the mean differences between the scans was reduced. Also the shift of the difference was reduced and was found to be $0.19[\mathrm{s}^{-1}]$. The coefficient of variation was calculated to be 3.62%.

S4 Fly-Through Animations

R_2 maps of all six subjects are available as fly-through animations on our repository https://schmidt-jo.github.io/mese_t2_paper/.
